# The Promoter Toolbox for Recombinant Gene Expression in *Trichoderma reesei*

**DOI:** 10.3389/fbioe.2018.00135

**Published:** 2018-10-11

**Authors:** Elisabeth Fitz, Franziska Wanka, Bernhard Seiboth

**Affiliations:** ^1^Research Division Biochemical Technology, Institute of Chemical, Environmental and Bioscience Engineering, TU Wien, Vienna, Austria; ^2^Austrian Centre of Industrial Biotechnology (ACIB) GmbH, Institute of Chemical, Environmental and Bioscience Engineering, TU Wien, Vienna, Austria

**Keywords:** constitutive promoter, inducible promoter, cellulase, promoter engineering, recombinant protein production, synthetic biology, strain engineering, *Trichoderma reesei*

## Abstract

The ascomycete *Trichoderma reesei* is one of the main fungal producers of cellulases and xylanases based on its high production capacity. Its enzymes are applied in food, feed, and textile industry or in lignocellulose hydrolysis in biofuel and biorefinery industry. Over the last years, the demand to expand the molecular toolbox for *T. reesei* to facilitate genetic engineering and improve the production of heterologous proteins grew. An important instrument to modify the expression of key genes are promoters to initiate and control their transcription. To date, the most commonly used promoter for *T. reesei* is the strong inducible promoter of the main cellobiohydrolase *cel7a*. Beside this one, there is a number of alternative inducible promoters derived from other cellulase- and xylanase encoding genes and a few constitutive promoters. With the advances in genomics and transcriptomics the identification of new constitutive and tunable promoters with different expression strength was simplified. In this review, we will discuss new developments in the field of promoters and compare their advantages and disadvantages. Synthetic expression systems constitute a new option to control gene expression and build up complex gene circuits. Therefore, we will address common structural features of promoters and describe options for promoter engineering and synthetic design of promoters. The availability of well-characterized gene expression control tools is essential for the analysis of gene function, detection of bottlenecks in gene networks and yield increase for biotechnology applications.

## Introduction

*Trichoderma reesei* is a model organism for plant biomass degradation and a production platform for proteins and enzymes (Bischof et al., 2016). The *T. reesei* reference strain QM6a was isolated during the Second World War on the Solomon Islands due to its ability to degrade US army tent canvas. Initial efforts to understand the biochemical mechanisms of its extracellular cellulases were performed by Mary Mandels and Elwyn T. Reese in the Natick Army Research Laboratories (Reese, 1976; Allen et al., 2009). The isolated wild-type strain *T. reesei* QM6a is, as many naturally occurring strains, a poor cellulase producer but showed a high potential for enhancement of secretion capacity. Therefore, it was necessary to run several strain improvement programs to convert QM6a into an industrial workhorse for cellulase production. These efforts resulted in many different mutagenized strains with the two main lineages from Rutgers and Natick (Reese, 1975; Montenecourt and Eveleigh, 1979). In the 1980ties the Rutgers lineage strain Rut-C30 (Peterson and Nevalainen, 2012) produced and secreted 30 g protein per liter fermentation medium using lactose as inducing carbon source (Durand et al., 1988). Further advancements by classical and genetic strain improvement led to industrial hyperproducer strains which are able to secrete more than 100 g of protein per liter in industrial fermentation settings, the new golden standard for cellulase production (Cherry and Fidantsef, 2003). Since all improved strains are derived from the single Solomon Islands isolate QM6a, a genomic tracking of changes accompanied with cellulase hyperproduction is possible (Le Crom et al., 2009). The potential to secrete huge amounts of protein promoted efforts to use *T. reesei* as expression host for recombinant proteins. Following the establishment of transformation techniques (Penttilä et al., 1987; Gruber et al., 1990), numerous fungal and non-fungal proteins were successfully produced by *T. reesei* (Nevalainen and Peterson, 2014; Paloheimo et al., 2016). Among these, calf chymosin was one of the first mammalian proteins produced in fungi (Harkki et al., 1989). Another emerging application is the biosynthesis of human proteins. Although *T. reesei* is not able to produce proteins with a human like glycosylation pattern, one advantage is that it does not hyperglycosylate proteins. These high-mannose glycans are often observed in proteins from other fungal expression hosts including *Saccharomyces cerevisiae* or *Pichia pastoris* and affect protein function or lead to immunogenic reactions in patients when used as therapeutic proteins (van Arsdell et al., 1987; Penttilä et al., 1988; Godbole et al., 1999; Boer et al., 2000; Jeoh et al., 2008; Ward, 2012). Promising results were recently achieved for a number of human proteins including antibodies, interferon alpha 2b, and insulin like growth factor when expressed in multiple protease deficient strains (Landowski et al., 2015). Besides its excellent yield of extracellular proteins, some of the secreted enzymes of *T. reesei* received the GRAS status (Generally Recognized as Safe) by the US Food and Drug Administration (U.S. Department of Health and Human Services, www.fda.gov). Another advantage is the cultivation on inexpensive media and the possibility for upscaling to reactor volumes larger than 100 m^3^ without compromising productivity (Paloheimo et al., 2016). *T. reesei* can grow on cheap lignocellulosic waste materials from food and non-food crops as easy available carbon. The lignocellulolytic enzymes produced under these conditions are then used to convert plant cell wall polysaccharides into simple fermentable sugars which are microbiologically transformed into bioethanol or other biorefinery products with higher value (Belal, 2013; Saravanakumar and Kathiresan, 2014; Xu et al., 2015).

For recombinant protein expression, the existence of a well-developed genetic toolbox is essential for efficient engineering of the production host. Its main parts were extensively reviewed, for example in (Keränen and Penttilä, 1995; Steiger, 2013; Bischof and Seiboth, 2014). The basic requirements are suitable host strains and preferentially a large set of different gene expression cassettes. One important element of an expression cassette is the promoter, which initiates and controls gene expression.

In this review, we will describe established promoters for *T. reesei* with emphasis on their advantages and disadvantages and discuss recently discovered promoters using transcriptomic approaches. Subsequently, we will give examples for other useful promoters that could be adapted for *T. reesei* and describe synthetic expression systems that have recently been or are about to be established for *T. reesei*. Finally, we will touch the emerging field of promoter engineering, where we will address the promoter architecture in eukaryotes, specific features of promoters and transcription factors in *T. reesei* and give an outlook on the *in silico* design of promoters.

## Established promoters for gene expression in *T. reesei*

Promoters are regulatory regions upstream of the transcription start site controlling the transcription of genes. They provide information for the binding of RNA polymerase and factors necessary for recruitment of the RNA polymerase. Initiation of transcription is regulated by a diverse set of activators and repressors with often further coactivators or corepressors involved (Wei et al., 2011). Although our knowledge on fungal promoters is steadily increasing, it is still considerable low compared to prokaryotic promoters and the exact composition of regulatory motives found in promoters of eukaryotes is still not sufficiently characterized. Some of the known features are summarized in section Structure and Regulation of Promoters.

**Figure 1:**
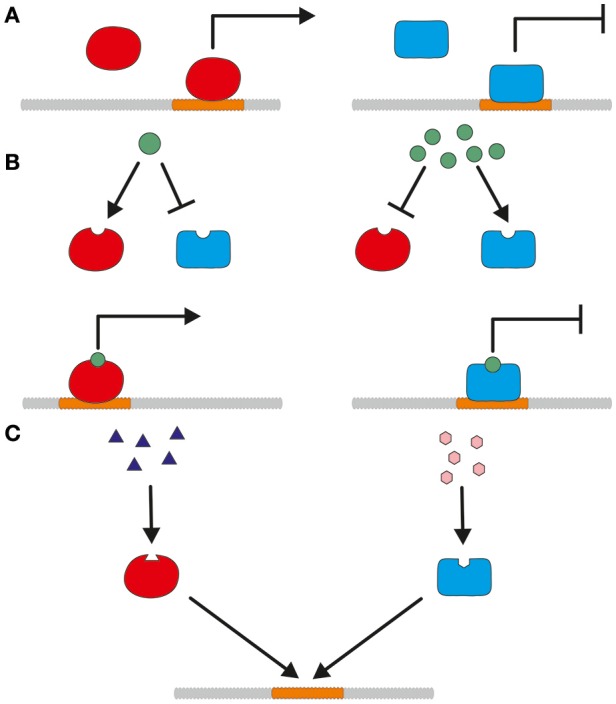
A simplified model for the different mode of actions of constitutive and tunable promoters. **(A)** Constitutive expression of a promoter is regulated by activation (left) and repression (right) without the influence of biotic or abiotic factors. **(B)** Concentration-dependent activation or repression of promoter regulation. Depending on the concentration, the modulating substance influences the action of transcriptional activators and repressors. **(C)** Transcriptional regulation by competitive activation and repression. Transcriptional activators and repressors bind to the promoter and compete for *cis*-regulatory binding sites.

Promoters can be classified into constitutive and tunable promoters. Constitutive promoters are expressed independently of environmentally induced transcription factors. Figure 1A shows such an independent activation or repression. In contrast to constitutive promoters, tunable promoters react to presence or absence of biotic or abiotic factors as shown in Figures 1B–D. Bidirectional promoters are a special case and able to regulate two adjacent genes oriented in opposite directions. They can be employed to express two genes simultaneously but will not be further addressed in this review, since they are not yet broadly used for *T. reesei*.

### Constitutive promoters

Constitutive promoters regulate expression of basal genes, like housekeeping genes or genes of the glycolytic pathway. They produce at a constant rate independent of the employed carbon source resulting in about the same amount of gene product over time. However, their activity is not very flexible, since they lack an on/off option. Constitutive promoters are independent of environmental factors. Nevertheless, they are often correlated to the growth of the fungus. Using the term constitutive is not completely correct in that context. The here described promoters can also be considered as auto-inducible, since their independence from growth factors has not yet been shown. The term auto-inducible is often used with bacterial expression hosts as *Escherichia coli* (Briand et al., 2016; Anilionyte et al., 2018). In this review, we will stick to the term “constitutive” as it is still generally used in literature. The most commonly used constitutive promoters for *T. reesei* were usually only tested under specific conditions for protein production. Especially d-glucose based media are cheap and therefore attractive for economic protein production. Thus, it would need further investigations to figure out, whether they are truly independent of media components or if they are active during all growth phases.

Frequently used homologous constitutive promoters are listed in Table 1. The promoters of *cDNA1* (Nakari-Setälä and Penttilä, 1995) and *tef1* (Nakari et al., 1993) were isolated by screening of cDNA libraries for genes highly expressed during growth on d-glucose and were used for overexpression in d-glucose containing media. The promoter of the uncharacterized *cDNA1* is generally regarded as one of the strongest among the constitutive promoters, while P*tef1* is medium strong (Nakari-Setälä and Penttilä, 1995; Uzbas et al., 2012). Other promoters of *eno1, gpd1* and *pdc1* are also well expressed during cultivation on d-glucose (Chambergo et al., 2002; Li et al., 2012). (Li et al., 2012) compared their activities by expression of the *T. reesei* xylanase *xyn2*. They found that P*pdc1* and P*eno1* are stronger than P*gpd1*, but that P*gpd1* showed a very stable expression, whereas P*pdc1* and P*eno1* activities increased on high d-glucose concentrations in the medium (Li et al., 2012). The homologous cellobiohydrolase *cel7a* was overexpressed under P*eno1* control in a Δ*cel7a* strain resulting in suitable product levels (Linger et al., 2015). The promoter of *pki1* was used for e.g., xylanase production exhibiting low to medium strength (Kurzatkowski et al., 1996).

**Table 1:** Examples for constitutive promoters for *T. reesei*.

**Promoter**	**Gene function**	**Remarks**	**References**
*cDNA1*	Unknown	Strong constitutive promoter in *T. reesei*, commonly used	Nakari-Setälä and Penttilä, 1995
*eno1*	Enolase	Activity enhances with increasing d-glucose concentration	Li et al., 2012
*gpd1*	Glyceraldehyde-3-phosphate dehydrogenase	Stable activity on d-glucose	Li et al., 2012
*pdc1*	Pyruvate decarboxylase	Activity enhances with increasing d-glucose concentration	Li et al., 2012
*pki1*	Pyruvate kinase	Medium strength	Kurzatkowski et al., 1996
*tef1*	Transcription elongation factor 1α	Medium strong, commonly used constitutive promoter	Nakari-Setälä and Penttilä, 1995
*rp2*	Ribosomal protein	Expression feasible	He et al., 2013

Overall, the comparison of the expression strength of these different promoters is difficult as the cultivation conditions, media compositions or strain backgrounds vary considerably in the different publications. So far, no results are available, which compare these promoters under industrial production conditions. Although constitutive promoters are simple to use and independent of media requirements, the major disadvantage in comparison to inducible promoters is their expression strength. The strong constitutive *cDNA1* promotor produced significantly less protein in a cultivation using d-glucose compared to the strong inducible *cel7a* promoter under cellulase inducing conditions (Penttilä et al., 1987).

### Tunable promoters

Tunable promoters are either inducible or repressible and dependent on the presence or absence of activating or repressing agents. These can be substances like sugars, amino acids, vitamins, metals or physical stimuli like light or different temperatures. The different mode of action of substance related activation and repression is depicted in Figures 1B,C. The effect of these modulating substances can be e.g., concentration dependent or competitive. Preferably, the strength of the promoter can be fine-tuned by the addition of different quantities of the inducing/repressing substance. For protein production, an inducible promoter should have no or only a low basal expression which is considerably enhanced by addition of the inducer. The induction should be strong, but in contrast to gene function studies a full tightness is not the main concern but surely advantageous to separate growth and production phase (Meyer et al., 2011; Huang et al., 2015). In both cases, the induction can be the result of a direct activation and the neutralization of a repressor, respectively. The expression level of a repressible promoter should be high to moderate and significantly lowered by addition of a repressing substance. As a production host for cellulases and xylanases, the promoters of genes of the main secreted enzymes are attractive targets for use in overexpression cassettes.

#### Cellulase promoters

The cellulase cellobiohydrolase CBH1, or according to the Carbohydrate-active Enzymes (CAZyme) annotation CEL7A (Lombard et al., 2014), constitutes with about 60% the dominating protein in the *T. reesei* secretome produced under cellulase inducing conditions. Its expression strength has made the *cel7a* promoter the first choice to drive recombinant protein production (Gritzali and Brown, 1979; Nummi et al., 1983). *Cel7a* promoter based expression constructs can be introduced in multiple copies. It is estimated that up to four copies of the expression cassette can still increase protein production but that at higher copy numbers a saturation effect occurs which is assumed to be caused by a depletion of transcriptional activators (Karhunen et al., 1993; Margolles-Clark et al., 1996). Besides the one of *cel7a*, there are a few other frequently used cellulase promoters listed in Table 2, including P*cel6a* or P*egl2*. Beside their lower expression strength, all of them exhibit similar advantages and limitations as the promoter of *cel7a*.

**Table 2:** Examples for tunable promoters for *T. reesei*.

**Cate- gory**	**Gene**	**Gene function**	**Inducible with**	**Repressible with**	**References**
Cellulase	*cel6a*	Cellobiohydrolase CBH2/CEL6A	Cellulose/wheat straw, sophorose, lactose	d-glucose	Zeilinger et al., 1998, 2003; Rahman et al., 2009; Wang and Lu, 2016
	*cel7a*	Cellobiohydrolase CBH1/CEL7A,	Cellulose/wheat straw, sophorose, lactose	d-glucose	Nevalainen et al., 2005; Uzbas et al., 2012; Li et al., 2017
	*cel5a*	Endoglucanase EG2/CEL5A	Cellulose/wheat straw, sophorose, lactose	d-glucose	Miyauchi et al., 2013
	*cel12a*	Endoglucanase EG3/CEL12A	Cellulose/wheat straw, sophorose, lactose	d-glucose	Rahman et al., 2009
Xylanase	*xyn1*	Xylanase XYN1	Xylan, d-xylose (conc. dependent)	d-glucose, d-xylose (conc. dependent)	Zeilinger et al., 1996; Mach-Aigner et al., 2010; Pucher et al., 2011; Nakazawa et al., 2012; Herold et al., 2013
	*xyn2*	Xylanase XYN2	Xylan, d-xylose (conc. dependent), xylobiose, cellobiose, sophorose	Partly d-glucose, d-xylose (conc. dependent)	Zeilinger et al., 1996; Mach-Aigner et al., 2010; Zhang et al., 2018
	*xyn3*	Xylanase XYN3	Cellulose, l-sorbose, sophorose	d-glucose, d-xylose (conc. dependent)	Xu et al., 2000; Rahman et al., 2009; Nakazawa et al., 2012; Hirasawa et al., 2018
Sugar trans-porter	*stp1*	Sugar transporter	d-glucose	Not investigated	Ward, 2011; Zhang et al., 2013, 2015
Various	*tauD3*	tauD like dioxygenase	–	l-methionine	Bischof et al., 2015
	Gene ID 70383	Dehydrogenase	Pantothenic acid	–	Gamauf et al., 2018
	*tcu1*	Copper transporter	Copper depletion	Copper addition	Wang et al., 2018

Cellulase genes have to be induced by sugars, which need to be present in the fermentation medium. Consequently, the application of those promoters is limited by the media requirements. Induction of cellulase promoters can be achieved by a broad variety of carbohydrates, ranging from insoluble polymeric carbon sources like cellulose and cellulose containing raw materials, to soluble disaccharides. Some of the best explored and most used inducing carbon sources can be found in Table 3. Cellulose and related carbon sources can be disadvantageous for industrial processes due to the insolubility of the substrate, which can affect downstream processing. The soluble disaccharide sophorose is too expensive for industrial use and lactose leads to lower enzyme production than cellulose in most strains. The monosaccharide l-sorbose affects growth of the fungus and is usually not used in industrial protein production. A more detailed discussion on further aspects of these inducing carbon sources was reviewed before (Stricker et al., 2008a; Kubicek et al., 2009; Amore et al., 2013).

**Table 3:** Inducing substrates for cellulase and xylanase expression.

**Induction of**	**Type of carbohydrate**	**Carbon source**	**Expression level/comment**	**References**
Cellulases	Polysaccharide	Cellulose	Insoluble, high induction	Stricker et al., 2008a; Kubicek et al., 2009; Amore et al., 2013.
	Polysaccharide	Wheat straw	Insoluble, raw plant material, high induction	Bischof et al., 2013
	Disaccharide	Sophorose	Expensive carbon source, high induction	Mandels et al., 1962; Vaheri et al., 1979; Suto and Tomita, 2001
	Disaccharide	Lactose	Medium induction	Mandels and Reese, 1957; Ivanova et al., 2013
	Monosaccharide	l-sorbose	Affects growth of the fungus, high induction	Kawamori et al., 1986; Nogawa et al., 2001
Xylanases	Polysaccharide	xylan	Insoluble, high induction	Zeilinger et al., 1996; Mach and Zeilinger, 2003
	Disaccharide	xylobiose	Soluble degradation product from xylan, consisting of two xylose-monomers	Mach and Zeilinger, 2003; Herold et al., 2013
	Monosaccharide	d-xylose	Induction at low concentrations, at high concentrations repression mediated by CRE1	Zeilinger et al., 1996; Mach-Aigner et al., 2010; Herold et al., 2013
	Monosaccharide	l-arabinose, l-arabitol	Induction at low concentrations	Mach-Aigner et al., 2011; Herold et al., 2013

Cellulase promoters, including P*cel7a*, are usually inactive during growth on d-glucose and other easily metabolizable carbon sources, such as d-xylose, due to a mechanism termed carbon catabolite repression (Ruijter and Visser, 1997; Mach-Aigner et al., 2010). In the cellulase hyperproducer strain *T. reesei* Rut-C30, ancestor of many industrial strains, carbon catabolite repression was abolished and therefore the strain can express cellulase and xylanase genes on d-glucose and other non-inducing carbon sources. This is the result of a mutation in the carbon catabolite repressor gene *cre1* (Ilmén et al., 1996b; Seidl et al., 2008). However, these derepressed cellulase levels are considerably lower compared to the induced expression levels (Nakari-Setälä et al., 2009).

A side effect when using cellulase promoters for protein expression is that induction leads to the expression of other native cellulases. This does not constitute a problem when the whole protein mixture is used for e.g., plant biomass degradation but leads to a loss of energy for the production and secretion of these byproducts in case a pure product is desired. In addition, they lead to increased costs in the downstream processing during protein purification. These problems are partially prevented by knocking out the most prominent cellulases (Landowski et al., 2015) or targeting the expression cassette to the *cel7a* locus to eliminate formation of the major cellobiohydrolase CEL7A. As an alternative, (Uzbas et al., 2012) established a generally cellulase and xylanase free production platform in *T. reesei* by using a strain deleted in the main cellulase activator XYR1. By doing so, the enzyme activities can be determined directly in the supernatant without disturbance of native cellulases or xylanases avoiding laborious purification steps. This system was already used to express various proteins, for example the *T. reesei* swollenin SWO1 and a *Corynascus thermophilus* cellobiose dehydrogenase (Eibinger et al., 2016; Ma et al., 2017).

#### Alternative tunable promoters

Whereas most cellulases are coordinately regulated, the expression of the xylanases can differ to some extent. There are several xylanase promoters in use (Table 2). Xylanases are induced by sugars, but in contrast to cellulases, not all of them are induced and repressed by the same substances. XYN1 and XYN3 are repressed by CRE1, respectively d-glucose, while XYN2 retains a low constitutive expression on d-glucose (Mach-Aigner et al., 2010; Herold et al., 2013). XYN2 is also induced by cellulose and related inducers (Amore et al., 2013; Herold et al., 2013). The most common used xylanase promoters are the ones of *xyn1* and *xyn2*.

Whereas cellulase and xylanase promoters are repressed on d-glucose, the promoter of the sugar transporter *stp1* is turned on when d-glucose is used as carbon source. Promising studies have been made for this alternative promoter, that can use cheap d-glucose but also other carbon sources as activating agent (Ward, 2011; Zhang et al., 2013, 2015).

## New developments to expand the promoter toolbox

Rational strain engineering demands novel regulatory tools to understand the complexity, limitations and driving forces of protein production. Therefore, it is necessary to regulate genes in a new manner as simply overexpression or deletion will not be sufficient to grasp the complexity of protein production. Nowadays, whole gene networks need to be adjusted in order to optimize product formation. Common obstacles can for example be metabolite pool drownings, unfavorable changes in the redox state of the cell, protein misfolding or protease formation caused by overexpression of recombinant proteins. Furthermore, complex gene systems often compensate for simple genetic modifications and cannot be characterized by only silencing or activating a single gene. All these drawbacks can be overcome by directed changes in expression or by construction of new gene circuits.

As detailed in the previous chapters, there is an adequate selection of promoters available for recombinant protein expression derived from cellulase or xylanase genes, respectively a few constitutive promoters with medium strength. However, they have a number of limitations. One of the problems of these inducible promoters is that they are controlled by a similar set of transcription regulators including the activator XYR1 and repressor CRE1 and that the inducing substrates lead to the expression of other coregulated enzymes and proteins. In addition, introduction of multiple copies of the *cel7a* promoter influences the expression of *cel7a* and other members of this regulon (Karhunen et al., 1993). Consequently, only very limited options for rational strain engineering apart from cellulase induction are possible. Therefore, it is necessary to establish new promoters and develop synthetic expression systems, which are regulated independently from the carbon source and in a cellulase and xylanase independent fashion. Furthermore, the industrial processes in the bioethanol or biorefinery industry demand more and more the utilization of lignocellulosic waste material from food and non-food crops as cheap carbon sources for the production of bioethanol or biorefinery products (Seidl and Seiboth, 2010). Ideally, the respective gene modulating substances should not be present in the fermentation medium, which is often difficult to achieve when such complex carbon sources as wheat straw are used.

The advent of genomics and transcriptomics facilitates the discovery of new promoters with new characteristics and qualities. In theory, there are hundreds of native tunable promoters available for each production host, whose expression strength can vary with biotic and abiotic substances available in the medium, growth state or developmental phase. State-of-the-art identification of new potential promoters and inducer or repressor are comparative transcriptomic analyses using either microarray or the generally more accurate RNA-Seq (Wang et al., 2009; Nazar et al., 2010). A further advantage of this approach is that it is not only possible to identify new suitable promoters but it is also possible to estimate how strong the transcriptomic response is upon addition of the inducing or repressing substance. Preferentially only a minor set of genes should be affected to avoid changes in product formation or growth. Therefore, some of the gene expression modulating substances described below were specifically tested for their effect on gene transcript levels expression during growth on the raw plant biomass wheat straw as carbon source. With this approach also published expression data can be analyzed as shown for example for *Aspergillus niger* to identify constitutive promoters (Blumhoff et al., 2013). Likewise, comparative genomics enhances the identification of orthologous sequences and accelerates thereby the transferability of promoters and the adaptation of expression systems from related fungi to *T. reesei*, as further source for new genetic tools.

### New tunable promoters discovered by transcriptomic studies

The assimilation of sulfur in microorganisms comprises genes that are also sensitive toward different sulfur containing substances such as l-methionine. *MET*/*met* genes offer a set of promoters of medium strength that are tightly repressed in the presence of l-methionine. The efficient inactivation of the most commonly used promoter of the l-methionine repressible ATP sulphurylase *MET3* was demonstrated for different yeasts including *Saccharomyces cerevisiae* (Cherest et al., 1985; Mao et al., 2002), *P. pastoris* (Delic et al., 2013), *Candida albicans* (Care et al., 1999), and *Ashbya gossypii* (Dünkler and Wendland, 2007). Addition of l-methionine to the medium does not interfere with *T. reesei* biomass formation or native cellulase production making this amino acid a suitable substance to repress gene expression (Gremel et al., 2008; Bischof et al., 2015). The use of different orthologues of these *met* genes as source for repressible promoters was therefore also tested for *T. reesei*. Although the *MET3* orthologue in *T. reesei* is highly repressible when simple cellulase inducing carbon sources as lactose were used, *met3* repression was absent in the presence of the complex carbon source wheat straw in the medium. Therefore, it was necessary to identify new l-methionine repressible genes. Transcriptomic comparison of cultures grown on wheat straw treated with and without l-methionine revealed that only 50 genes were differentially regulated upon l-methionine addition. Among the down-regulated genes, a promoter of *tauD* like dioxygenases was successfully tested with the invertase *sucA* from *Aspergillus niger* as reporter. A strong repression of invertase expression was found in wheat straw cultures, even hours after addition of l-methionine. This repressible promoter is not restricted to wheat straw cultures and was also functional during growth on other carbon sources including d-glucose and glycerol (Bischof et al., 2015).

Analogous to l-methionine repressible promoters, new inducible promoters were discovered which do not interfere with cellulase production or growth of *T. reesei*. Among different amino acids and vitamins tested, pantothenic acid was identified as suitable inducer at low substrate concentrations. Comparative microarray analysis between a pantothenic induced and a mock treated wheat straw culture identified only a small number of genes that were differentially regulated. Six of the highest inducible genes were found in a gene cluster including a putative pantothenic acid transporter. Fusion of promoters of these pantothenic acid inducible genes to the *T. reesei* ß-glucosidase BGL1 showed a clear induction of expression at low amounts with 0.1 and 1 mM pantothenic acid (Gamauf et al., 2018).

Another example for tuning of gene expression is the promoter of the copper transporter *tcu1*. Copper transporter are tightly repressed by environmental copper levels to efficiently regulate the uptake of copper. Copper responsive promoters were already successfully applied in other fungi (Ory et al., 2004; Lamb et al., 2013) and were recently adapted for *T. reesei* (Lv et al., 2015)*. T. reesei tcu1* is the orthologue of the *N. crassa* copper transporter and its activity depends on the copper concentration in the medium but is independent of the carbon source. Its expression is abolished if a certain amount of copper is present in the medium and can be relieved by the addition of a Cu^2+^ chelator. The function of the *tcu1* promoter was tested by expressing the cellulase and xylanase activator XYR1. Using this promoter a derepression of cellulase production in *T. reesei* was achieved by *xyr1* expression (Lv et al., 2015). The promoter was further engineered to be more sensitive toward lower copper concentrations in the μM scale and was established in the context of an unmarked genetic modification strategy (Lv et al., 2015; Zhang et al., 2016b). The *tcu1* promoter was also applied to selectively silence genes without risking a lethal phenotype (Zheng et al., 2017; Wang et al., 2018), which qualifies the system for cost-effectively exploring functions of essential genes. An interesting question in the context of cellulase production is if the low copper concentrations can affect the activity of the cellulase cocktail as they contain beside the canonical glycoside hydrolases also copper-dependent oxidases.

An interesting option to control gene expression are developmentally regulated promoters. A set of genes active only under sporulation conditions was identified in *T. reesei* by a transcriptomic approach (Metz et al., 2011). These promoters could be used to express the proteins exclusively during the onset of sporulation and target the proteins to the conidiospores. In this case, the promoters do not need to be induced by addition of an agent to the medium, but are regulated by the developmental phase of the fungus.

### Examples for potential adaptation of promoters from other fungi to *T. reesei*

A number of promoters are found in other well-studied ascomycetes including *Neurospora* and *Aspergillus* spp. and could represent useful additions to the genetic toolbox of *T. reesei*.

#### Promoters from *Neurospora crassa*

An inducible promoter often used for *N. crassa* is the *qa-2* promoter which can be activated by quinic acid and which is repressed by d-glucose (Baum and Giles, 1985; Geever et al., 1987). The promoter works similarly in *T. reesei*, but is not yet optimized for gene expression, since it is highly sensitive to sugar concentrations in the culture (Zheng et al., 2017).

Beside chemically induced promoter, there are promoters regulated by a physical stimulus such as light. Light sensitive promoters are available but were tested only for a few organisms, for example *N. crassa* (Fuller et al., 2018). *T. reesei* ability to react to light stimuli was already the subject of a number of transcriptomic studies (Tisch et al., 2011), but a systematic search for light-sensitive promoters under different light conditions is still missing.

#### Promoters from *Aspergillus*

Several inducible promoters established for *Aspergillus* sp. show a great potential as alternatives for *T. reesei*. For example, the recently described promoter of *bphA* (benzoate para-hydroxylase) from *A. niger* can be induced by the presence of benzoic acid. It is tightly repressed in the absence of benzoic acid and induced within 10 min upon its addition to the medium (Antunes et al., 2016). That the fungus can grow on benzoic acid as the sole carbon source indicates that toxicity is a minimal concern in this system.

There are also thiamine repressible *thiA* promoters available, which were tested successfully in *A. oryzae* und *A. nidulans*. One drawback is that they are not repressible under alkaline conditions (Shoji et al., 2005).

A challenge for protein production processes is to minimize the amount of carbon source necessary for biomass maintenance and formation but using the carbon source instead to maintain only high productivity. One possibility to uncouple product from biomass formation is to reach a specific growth rate close to zero. Whole genome transcriptomic analysis has identified a number of promoters active under these harsh conditions of zero growth for *Aspergillus* (Jørgensen et al., 2010; Wanka et al., 2016a), some of them are also available in *T. reesei*.

### Synthetic expression systems

**Figure 2:**
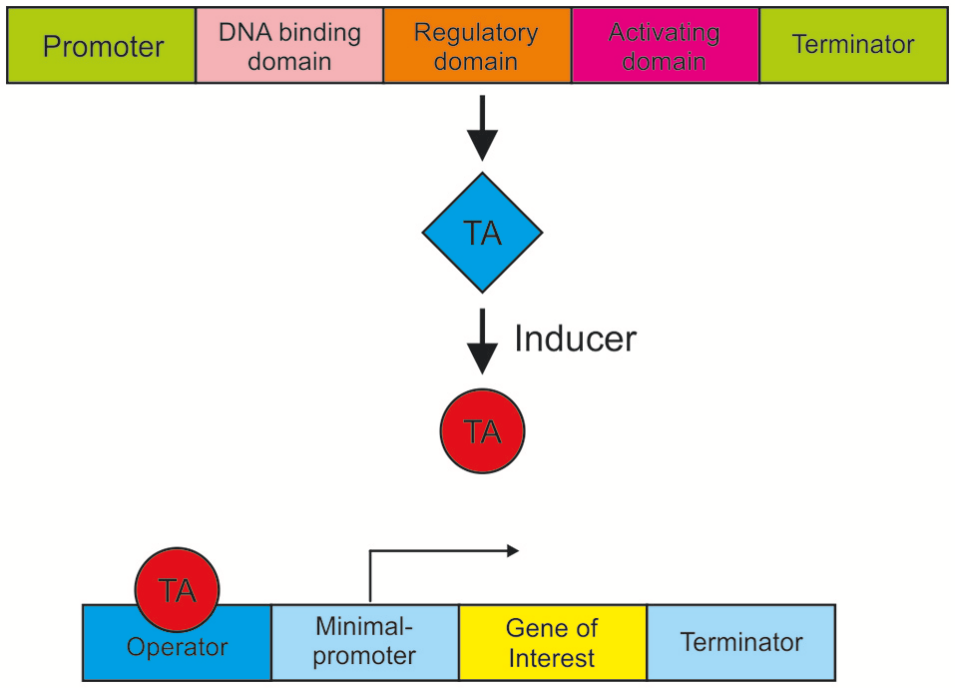
A schematic presentation of synthetic expression system for gene activation. A promoter drives the expression of a synthetic transcription activator (TA) containing a DNA binding domain (e.g., LexA or GAL4), a regulatory domain (e.g., an estrogen receptor) and a transcription activation domain (e.g., VP16). Upon binding of an inducer the conformation of the transactivator changes and interacts with the operator that initiates the transcription of the gene of interest.

In order to realize a metabolism-independent expression and accurate regulation of genes, synthetic expression systems are recommended. Such a well-engineered expression system should respond in a controlled manner to input, for example to an inducing agent, be tightly regulated, cover different expression strength and be non-toxic. In most cases, the controlled gene expression is achieved by the use of a synthetic transactivator, which consists of a fusion of different protein domains. Synthetic transactivators comprise a DNA-binding domain, a heterologous regulatory domain and an activation domain e.g., derived from the *Herpes simplex* virus protein 16 (VP16), as shown in Figure 2. This transactivator usually requires a chemical ligand for binding to the responsive operator sequence. To increase gene expression multiple copies of the operator are usually introduced to the promoter. The goal is to establish a variety of regulatory systems in *T. reesei* to simultaneously but independently regulate or balance the expression of different genes. Figure 2 shows the general mode of action of such an expression system. Examples for such expression systems are the estrogen receptor system, a light regulated expression system and the Tet-on/off system.

The inducible estrogen receptor system (hERα) was successfully applied for *S. cerevisiae*. It is based on the human estrogen receptor hERα, a member of a family of nuclear receptors for small hydrophobic ligands. The hERα was fused to the DNA-binding domain of LexA or of *S. cerevisiae* Gal4 and the VP16 activation domain (Quintero et al., 2007; Ottoz et al., 2014; Dossani et al., 2018). The expression system was successfully tested in *Aspergillus nidulans* and *A. niger*, in which diethylstilbestrol or 17-β-estradiol regulated hERα to activate reporter gene transcription via estrogen-responsive elements (Pierrat et al., 1992; Pachlinger et al., 2005). A downside of the system is that it is either highly inducible but exhibits a high basal expression level or it is tightly regulated but only weakly inducible (Pachlinger et al., 2005; Meyer et al., 2011). A respective system from plants (Zuo et al., 2000) was adapted to *T. reesei* with similar results (Derntl, 2018).

Controlling the expression strength of genes with light promises a variety of advantages upon regulation with chemical agents: (i) light can be applied and removed in an instance, (ii) it is cheap and simple to obtain, (iii) it is easy to control intensity and duration, that means it is fully tunable and (iv) no specific media composition is required. A main drawback is, however, to implement this system on an industrial scale where high biomass concentrations pose clearly a challenge. In *S. cerevisiae* light sensitivity toward blue or red light was introduced by fusing photoreceptors of *Arabidopsis* to the *S. cerevisiae GAL1* promoter (Hughes et al., 2012). (Wang et al., 2014) developed a light-sensitive transactivator for *T. reesei* by fusing the *S. cerevisiae* Gal4 DNA-binding domain to the *N. crassa* blue-light photoreceptor Vivid and the *H. simplex* VP16 activation domain (Zoltowski et al., 2007). The transactivator was then set under the control of the *pki1* promoter and the expression of two reporter genes was successfully induced by light pulses (Wang et al., 2014). This “expression-switch” can be inserted to commonly used promoters to make them light-sensitive (Zhang et al., 2016a).

An almost universal applied expression system is the Tet-on/off system (Gossen and Bujard, 1992). In case of the Tet-on system the transcription is dose-dependently activated by the addition of the synthetic tetracycline derivative doxycycline, which bind to the reverse transactivator rtTA. This complex then binds to the *tet*O operator sequence activating the transcription of the gene of interest. The Tet-off system consists of the tetracycline-controlled transactivator tTA, which allows permanent expression of the gene of interest in the absence of tetracycline. Addition of tetracycline prevents tTA binding to the *tetO* operator and represses thereby expression. This systems have been adapted for fungi including *A. fumigatus* (Vogt et al., 2005; Helmschrott et al., 2013) and *A. niger* (Meyer et al., 2011; Wanka et al., 2016b), but obstacles are observed for a successful adaptation for *T. reesei* (Zheng et al., 2017).

Overall, the selection of established artificial expression systems for *T. reesei* is very limited. However, there are multiple systems available, which show high potential. Examples include the temperature inducible gene regulation (TIGR) system (Weber et al., 2003), cumate gene-switch (4-isopropylbenzoic acid) (Mullick et al., 2006), biotin triggered genetic switch (Weber et al., 2009), pristinamycin on/off system (Fussenegger et al., 2000) or erythromycin on/off system (Reeves et al., 2002).

Expression systems are usually only adapted for one specific host organism and can be transferred effectively only to a limited number of closely related species. Therefore, (Rantasalo et al., 2018) have developed a universal synthetic expression system for recombinant protein production for six different yeasts including *S. cerevisiae* and *P. pastoris* and the two fungi *T. reesei* and *A. niger*. The system comprises two different expression cassettes, including one which provides a weak constitutive level of a synthetic transcription factor (BM3R1-NLS-VP16) and a second one for strong tunable expression of the target gene via an synthetic transcription factor dependent promoter consisting of eight BM3R1 binding sites (Rantasalo et al., 2018).

## Engineering of promoters

**Figure 3:**
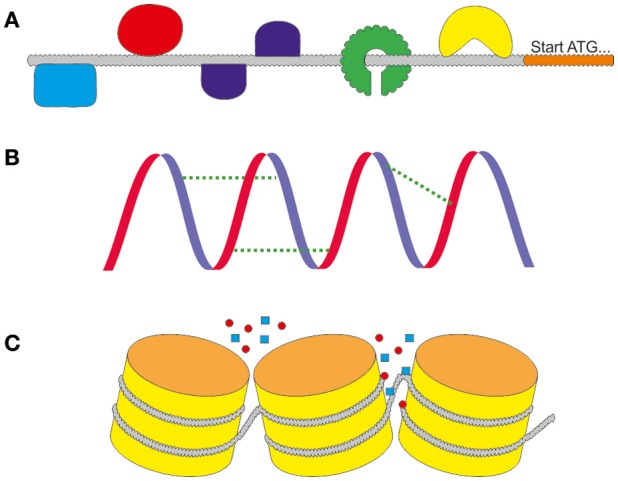
The different levels of regulation. **(A)** On the primary sequence the activity is regulated by the interaction with DNA binding molecules including as activating or repressing transcription factors (TF). They can bind in different ways to motifs in the sequence. **(B)** The activity is influenced by the secondary structure of the DNA. It can support or weaken the activity by stabilizing the primary sequence. **(C)** The tertiary structure of the chromatins regulates the accessibility of the promoter for DNA binding molecules.

The rational design of promoters is one of the newest fields within synthetic biology. The advantages are clear: a promoter with the needed characteristics can be designed independently of the natural spectrum of the production host. Ideally, the expression strength can be modulated, promoter switches can be added and inducibility or repression properties can be added or deleted. In the age of modern molecular biology, in which the synthesis of artificial DNA sequences is quick and cheap, the modification of primary sequences of promoters is a convenient way to optimize expression. The changes can alter different levels of regulation, as depicted in Figure 3 including transcription factor binding sites, secondary structure stabilities or chromatin accessibility. Sounds easy, but prior to the design of new promoters it is crucial to retrieve information about the sequences that lead to the desired characteristics. One backlash until today is that there are no adequate and reliable prediction software for promoter functions available. Neither for the modification of natural backbones, nor the rational design of completely synthetic promoters. Today, modified and synthetic promoters are still tested via trial and error.

### Structure and regulation of promoters

Regulatory elements of fungal promoters are still poorly characterized. However, a detailed knowledge of the binding motives in the promoters is necessary for rational engineering of promoters for biotechnological application.

The promoter consists of two merging parts: the core promoter which is responsible for its basal activity (Roeder, 1996; Roy and Singer, 2015) and the proximal promoter. The region near the start codon of the coding sequence contains the transcription start site for the RNA polymerase II (Smale and Kadonaga, 2003) and other enhancing elements (Novina and Roy, 1996). Usually they contain a TFIIB recognition site (Gelev et al., 2014), initiation site (Inr), motif 10 element (MTE); (Lim et al., 2004) and downstream promoter element (DPE) (Juven-Gershon and Kadonaga, 2010). The regulation of expression is determined by the accessibility of the DNA (Svejstrup, 2004; Müller and Tora, 2014), the RNA polymerase affinity respectively the stability of the initiation complex and transcription factors (Lemon and Tjian, 2000; Juven-Gershon and Kadonaga, 2010). The chromatin structure is another layer of regulation for the promoter. In eukaryotes, DNA can be tightly packaged with the help of histones forming the chromatin structure. This condensed state can prevent the RNA polymerase, activators and repressors to interact with the DNA. To allow access to the otherwise inactive DNA, chromatin remodeling is necessary to alter the architecture of these regions allowing transcriptional regulation by these factors. The relation between chromatin status and induction of cellulase production in *T. reesei* was already addressed in a number of publications (Seiboth et al., 2012; Mello-de-Sousa et al., 2015, 2016) but will not further be followed in this review.

### Modification of natural promoters

An obvious approach to alter the characteristics of a promoter is to add or delete transcription factor binding sites or genetic switches of the promoter (Mach and Zeilinger, 2003). Premise for this approach is that the respective *cis* acting sequences and mechanisms of activation/repression are already known. We will therefore shed light on the essential parts of eukaryotic promoter and on some of the known transcription factors and their binding sites specific for *T. reesei*.

A highly engineered promoter is the one of *cel7a*. By deleting CRE1 repression sites but leave induction sites intact, P*cel7a* can be used to express a recombinant gene on d-glucose, on which all native cellulases are usually repressed (Ilmén et al., 1996a). Several studies were made in which CRE1 binding sites were replaced by activator binding sites for e.g., ACE2 and HAP2/3/5 to abolish carbon catabolite repression and enhance activity of the engineered *cel7a* promoter (Liu et al., 2008; Zou et al., 2012). Other examples were already discussed above, like the insertion of sequences into the native promoter to add characteristics like light-inducibility or copper sensitivity. Multiple insertions of activating sites do not necessarily lead to increased promoter activity, as the general promoter architecture has to be considered, since the spacing between certain elements in the promoter can be play an important role as exemplified for the *cel7a* promoter (Kiesenhofer et al., 2017).

Another option to engineer promoters is the assembly of active sequences. This method is especially useful when the binding motifs and their interacting transcription factors are unknown. Natural promoter sequences with known expression characteristics or strength can be aligned and similar parts found among the sequences in these promoters can be divided into building blocks. These blocks can then be arranged in various ways to form synthetic promoters. Once a functional promoter is assembled, the building blocks can be further modified, duplicated or deleted to improve the overall activity of the new promoter (Hartner et al., 2008; Xuan et al., 2009; Mellitzer et al., 2014; Vogl et al., 2014). While this approach was already extensively used to optimize *S. cerevisiae* and *P. pastoris* promoters, it was not applied for *T. reesei* yet. An advantage of this system is that first hints for new transcription factor binding sites and their respective transcription factors can be found without prior knowledge.

#### General eukaryotic promoter elements

Although only present in 5–7% of all eukaryotic promoters, the TATA-box is one of the most known and well characterized core promoter elements (Roy and Singer, 2015). The TATA-box and the TATA-binding protein are necessary for assembly of the TATA-initiation complex (Smale and Kadonaga, 2003). For a detailed summary see (Yella and Bansal, 2017).

The HAP2/3/5-complex binding site or CAAT-box can be found in about 30% of eukaryotic promoters, more frequently in TATA-less promotors but also in promoters with a TATA-site (Mantovani, 1998). The HAP2/3/5-complex acts as enhancer and the presence of its binding motif CCAAT is necessary for high expression of *cbh2* (Zeilinger et al., 1998, 2001). The complex may play a role in the chromatin remodeling and therefore is involved in cellulase induction (Zeilinger et al., 2003).

CpG islands are mostly unmethylated, long stretches of CG-rich regions and are involved in the epigenetic regulation of transcription (Deaton and Bird, 2011). In such an island the GC content of a region of 200–2,000 bases (depending on the definition) is higher than 55% and it is thought that the GpC islands can initiate or silence gene expression, especially in promoters without TATA-box (Delgado et al., 1998; Chatterjee and Vinson, 2012). Several promoters can lie within one island. Most of the studies concerning the role of CpG islands were done in mammals but GpC islands can also be found in intergenic regions of the *T. reesei* genome. The regulatory role of such an element has not been investigated yet.

GC-box with its consensus sequence of GGGCGG is preferentially found in TATA-less promoters and is involved in the correct positioning of the RNA polymerase II (Weis and Reinberg, 1997).

#### *T. reesei* transcription factors and their binding sites

In *T. reesei* the best-studied transcription factors are involved in the regulation of cellulases. Recent research efforts demonstrate that cellulase regulation is controlled by a highly adapted regulatory network involving multiple transcription factors, which can directly or indirectly regulate cellulase expression. CRE1 is the major regulator for carbon catabolite repression. The transcription factor impairs the native cellulase production on glucose, which is mainly an indirect effect. During growth on d-glucose as carbon source, CRE1 mainly accounts for the repression of genes responsible for the entry of cellulase inducers into the cell. Studies have shown that the *cre1* gene is truncated in the industrial ancestor strain RUT-C30 and leads to a cellulase hyperproduction and derepression in that very strain (Strauss et al., 1995; Ilmén et al., 1996b; Takashima et al., 1996; Le Crom et al., 2009; Nakari-Setälä et al., 2009; Portnoy et al., 2011a). CRE1 binds to the SYGGRG sequence and it is believed that the functional binding sites consists of two closely spaced repeats (Cubero and Scazzocchio, 1994; Mach et al., 1996).

XYR1 is the master activator for cellulases and xylanases and is involved in d-xylose and l-arabinose catabolism. Lack of XYR1 completely eliminates cellulase expression (Stricker et al., 2008a; Akel et al., 2009; Furukawa et al., 2009; Portnoy et al., 2011b; Lichius et al., 2015). XYR1 binds to a GGCTAA sequence arranged as an inverted repeat (Stricker et al., 2006; Kiesenhofer et al., 2017) and seems to be regulated by a long non-coding RNA (Till et al., 2018).

ACE1 is a Cys_2_His_2_-type zing finger protein and binds to different AGGCA motifs (Saloheimo et al., 2000). A deletion of the transcription factor leads to an increased production of all (hemi-)cellulases, suggesting that it acts as a repressor for these class of enzymes (Aro et al., 2003).

ACE2 is a zinc binuclear cluster protein that binds to a GGCTAA sequence, which is the same binding motif as for XYR1 (Aro et al., 2001). A deletion of *ace2* leads to a significantly reduced expression of cellulases and biomass formation on cellulose. The induction time of *xyn1* and *xyn2* was reduced, but the overall xylanase production was lower in Δ*ace2* strains (Aro et al., 2001; Stricker et al., 2008b).

ACE3 is another activator. Its overexpression resulted in enhanced cellulase production, whereas the deletion impaired cellulase expression and severely decreased xylanase activity (Häkkinen et al., 2014). The exact binding motif is not yet determined.

RCE1 is a zinc binuclear cluster protein and acts as a transcriptional repressor. Lack of RCE1 facilitates cellulase induction and delays the termination of cellulase expression. RCE1 seems to antagonize the binding of XYR1 to the cellulase promoters (Cao et al., 2017).

Beside these, a number of other transcription factors have been characterized including ARA1, which is involved in the utilization of d-galactose and l-arabinose and regulates different CAZymes in response to d-galactose (Benocci et al., 2018). The xylanase regulator SxlR seems to be a repressor for GH11 family xylanases as an overexpression in RUT-C30 resulted in a reduced and a deletion in an increased xylanase activity (Liu et al., 2017). The deletion of the transcription factor Trpac1 lead to a significantly higher cellulase production at pH 7, but not at other pH values (He et al., 2014). For more detailed reviews concerning the transcriptional regulation of cellulases see (Kubicek et al., 2009; Bischof et al., 2016; Kunitake and Kobayashi, 2017) and (Benocci et al., 2017).

### Synthetic design of promoters

By gaining more and more insights into the regulation of expression, the way leads to the rational design of synthetic promoters optimized for the respective purpose. Several studies were made toward this direction in mammals (Juven-Gershon et al., 2006; Schlabach et al., 2010), but there are still a lot of unknown factors in gene expression and a lack of reliable prediction methods for an everyday application of rationally designed promoters. Due to this lack of deeper knowledge, a first approach for the design could be based on a Multivariate Data Analysis, in which the sheer number of potentially relevant factors can be statistically analyzed. In the next step, *in silico* assumptions have to be confirmed by experimental evaluation. The number of experiments can be drastically reduced by the Design of Experiment (DoE) approach. For example, a number of activating short sequences are identified and these elements are then arranged in different combination in a backbone. DoE will then help to reduce the number of actual sequences that have to be synthesized and tested. The activity of the new promoter sequences will then be determined and parts with higher or lower impact can be analyzed by Multivariate Data Analysis tools including PCA or PCR (Gagniuc et al., 2012). The designed promoters can further be optimized by additional rounds of engineering.

## Conclusion

For a long time, the natural low cellulase and xylanase production of *T. reesei* was enhanced for industry by random mutagenesis. To establish *T. reesei* as recombinant production host, it is more desirable to rationally engineer and optimize the strain for fermentation processes. Many tools are necessary for an efficient modification of the organism, one of them being a broad selection of promoters and expression systems. The choice of the respective promoter depends on the aim of the manipulation, for a simple overexpression a strong inducible promoter does the trick. For complex engineering issues, for example, the manipulation of metabolite pools or the alteration growth parameters toward more favorable characteristics in the fermenter, different promoters and expression systems are needed, that are not interfering with the protein production itself. Finally, synthetic biology offers new avenues and opens the possibility of *de novo* design of context-specific, customizable promoters. However, to realize these approaches in *T. reesei* and other fungi, it will be necessary to further increase our understanding of the regulatory networks governing gene expression.

## Author contributions

EF and BS prepared the outline of the manuscript. EF and FW wrote the main part of the manuscript. BS wrote the introduction, read and commented on the ms.

### Conflict of interest statement

The authors declare that the research was conducted in the absence of any commercial or financial relationships that could be construed as a potential conflict of interest.

## References

[B1] Akel E. Metz B. Seiboth B. Kubicek C. P. (2009). Molecular regulation of arabinan and l-arabinose metabolism in *Hypocrea jecorina* (*Trichoderma reesei*). Eukaryotic Cell 8:1837–1844. 10.1128/EC.00162-09 19801419 PMC2794218

[B2] Allen F. Andreotti R. Eveleigh D. E. Nystrom J. (2009). Mary Elizabeth Hickox Mandels, 90, bioenergy leader. Biotechnol. Biofuels 2:22. 10.1186/1754-6834-2-22 19723299 PMC2748067

[B3] Amore A. Giacobbe S. Faraco V. (2013). Regulation of cellulase and hemicellulase expression in fungi. Curr. Genomics 14, 230–249. 10.2174/1389202911314040002 24294104 PMC3731814

[B4] Anilionyte O. Liang H. Ma X. Yang L. Zhou K. (2018). Short, auto-inducible promoters for well-controlled protein expression in *Escherichia coli*. Appl. Microbiol. Biotechnol. 102:7007–7015. 10.1007/s00253-018-9141-z 29948110

[B5] Antunes M. S. Hodges T. K. Carpita N. C. (2016). A benzoate-activated promoter from *Aspergillus niger* and regulation of its activity. Appl. Microbiol. Biotechnol. 100, 5479–5489. 10.1007/s00253-016-7373-3 26907094

[B6] Aro N. Ilmén M. Saloheimo A. Penttilä M. (2003). ACEI of *Trichoderma reesei* is a repressor of cellulase and xylanase expression. Appl. Environ. Microbiol. 69, 56–65. 10.1128/AEM.69.1.56-65.2003 12513977 PMC152388

[B7] Aro N. Saloheimo A. Ilmén M. Penttilä M. (2001). ACEII, a novel transcriptional activator involved in regulation of cellulase and xylanase genes of *Trichoderma reesei*. J. Biol. Chem. 276, 24309–24314. 10.1074/jbc.M003624200 11304525

[B8] Baum J. A. Giles N. H. (1985). Genetic control of chromatin structure 5′ to the *qa-x* and *qa-2* genes of *Neurospora*. J. Mol. Biol. 182, 79–89. 10.1016/0022-2836(85)90029-4 3999144

[B9] Belal E. B. (2013). Bioethanol production from rice straw residues. Brazilian J. Microbiol. 44, 225–234. 10.1590/S1517-83822013000100033 24159309 PMC3804203

[B10] Benocci T. Aguilar-Pontes M. V. Kun R. S. Seiboth B. de Vries R. P. Daly P. (2018). ARA1 regulates not only l-arabinose but also d-galactose catabolism in *Trichoderma reesei*. FEBS Lett. 592, 60–70. 10.1002/1873-3468.12932 29215697

[B11] Benocci T. Aguilar-Pontes M. V. Zhou M. Seiboth B. De Vries R. P. (2017). Regulators of plant biomass degradation in ascomycetous fungi. Biotechnol. Biofuels 10:152. 10.1186/s13068-017-0841-x 28616076 PMC5468973

[B12] Bischof R. Fourtis L. Limbeck A. Gamauf C. Seiboth B. Kubicek C. P. (2013). Comparative analysis of the *Trichoderma reesei* transcriptome during growth on the cellulase inducing substrates wheat straw and lactose. Biotechnol. Biofuels 6:127. 10.1186/1754-6834-6-127 24016404 PMC3847502

[B13] Bischof R. Seiboth B. (2014). Molecular tools for strain improvement of Trichoderma spp., in Biotechnology and Biology of Trichoderma, eds Gupta V. G. Schmoll M. Herrera-Estrella A. Upadhyay R. S. Druzhinina I. Tuohy M. G. (Amsterdam: Elsevier), 179–191. 10.1016/B978-0-444-59576-8.00012-6

[B14] Bischof R. H. Horejs J. Metz B. Gamauf C. Kubicek C. P. Seiboth B. (2015). l-Methionine repressible promoters for tuneable gene expression in *Trichoderma reesei*. Microb. Cell Fact. 14:120. 10.1186/s12934-015-0308-3 26271614 PMC4536894

[B15] Bischof R. H. Ramoni J. Seiboth B. (2016). Cellulases and beyond: the first 70 years of the enzyme producer *Trichoderma reesei*. Microb. Cell Fact. 15:106. 10.1186/s12934-016-0507-6 27287427 PMC4902900

[B16] Blumhoff M. Steiger M. G. Marx H. Mattanovich D. Sauer M. (2013). Six novel constitutive promoters for metabolic engineering of *Aspergillus niger*. Appl. Microbiol. Biotechnol. 97, 259–267. 10.1007/s00253-012-4207-9 22707054

[B17] Boer H. Teeri T. T. Koivula A. (2000). Characterization of *Trichederma reesei* cellobiohydrolase Cel7a secreted from *Pichia pastoris* using two different promoters. Biotechnol. Bioeng. 69, 486–494. 10.1002/1097-0290(20000905)69:5<486::AID-BIT3>3.0.CO;2-N 10898858

[B18] Briand L. Marcion G. Kriznik A. Heydel J. M. Artur Y. Garrido C.. (2016). A self-inducible heterologous protein expression system in *Escherichia coli*. Sci. Rep. 6:33037. 10.1038/srep33037 27611846 PMC5017159

[B19] Cao Y. Zheng F. Wang L. Zhao G. Chen G. Zhang W.. (2017). RCE1, a novel transcriptional repressor, regulates cellulase gene expression by antagonizing the transactivator Xyr1 in Trichoderma reesei. Mol. Microbiol. 105, 65–83. 10.1111/mmi.13685 28378498

[B20] Care R. S. Trevethick J. Binley K. M. Sudbery P. E. (1999). The *MET3* promoter: a new tool for *Candida albicans* molecular genetics. Mol. Microbiol. 34, 792–798. 10.1046/j.1365-2958.1999.01641.x 10564518

[B21] Chambergo F. S. Bonaccorsi E. D. Ferreira A. J. S. Ramos A. S. P. Júnior J. R. F. Abrahão-Neto J.. (2002). Elucidation of the metabolic fate of glucose in the filamentous fungus *Trichoderma reesei* using expressed sequence tag (EST) analysis and cDNA microarrays. J. Biol. Chem. 277, 13983–13988. 10.1074/jbc.M107651200 11825887

[B22] Chatterjee R. Vinson C. (2012). CpG methylation recruits sequence specific transcription factors essential for tissue specific gene expression. Biochim. Biophys. Acta 1819, 763–770. 10.1016/j.bbagrm.2012.02.014 22387149 PMC3371161

[B23] Cherest H. Nguyen N. T. Surdin-Kerjan Y. (1985). Transcriptional regulation of the *MET3* gene of *Saccharomyces cerevisiae*. Gene 34, 269–281. 10.1016/0378-1119(85)90136-2 2989110

[B24] Cherry J. R. Fidantsef A. L. (2003). Directed evolution of industrial enzymes: an update. Curr. Opin. Biotechnol. 14, 438–443. 10.1016/S0958-1669(03)00099-5 12943855

[B25] Cubero B. Scazzocchio C. (1994). Two different, adjacent and divergent zinc finger binding sites are necessary for CREA-mediated carbon catabolite repression in the proline gene cluster of *Aspergillus nidulans*. EMBO J. 13, 407–415. 8313886 10.1002/j.1460-2075.1994.tb06275.x PMC394822

[B26] Deaton A. M. Bird A. (2011). CpG islands and the regulation of transcription. Genes Dev. 25, 1010–1022. 10.1101/gad.2037511 21576262 PMC3093116

[B27] Delgado S. Gómez M. Bird A. Antequera F. (1998). Initiation of DNA replication at CpG islands in mammalian chromosomes. EMBO J. 17, 2426–2435. 10.1093/emboj/17.8.2426 9545253 PMC1170585

[B28] Delic M. Mattanovich D. Gasser B. (2013). Repressible promoters - A novel tool to generate conditional mutants in *Pichia pastoris*. Microb. Cell Fact. 12:6. 10.1186/1475-2859-12-6 23347582 PMC3599224

[B29] Derntl C. (2018). Inducible expression of secondary metabolites using a synthetic transcription factor in an essentially background-free system, in 14th European Conference on Fungal Genetics (Haifa)

[B30] Dossani Z. Y. Reider Apel A. Szmidt-Middleton H. Hillson N. J. Deutsch S. Keasling J. D.. (2018). A combinatorial approach to synthetic transcription factor-promoter combinations for yeast strain engineering. Yeast 35, 273–280. 10.1002/yea.3292 29084380 PMC5873372

[B31] Dünkler A. Wendland J. (2007). Use of *MET3* promoters for regulated gene expression in *Ashbya gossypii*. Curr. Genet. 52, 1–10. 10.1007/s00294-007-0134-1 17479268

[B32] Durand H. Clanet M. Tiraby G. (1988). Genetic improvement of *Trichoderma reesei* for large scale cellulase production. Enzym. Microb. Technol. 10, 341–345.

[B33] Eibinger M. Sigl K. Sattelkow J. Ganner T. Ramoni J. Seiboth B.. (2016). Functional characterization of the native swollenin from Trichoderma reesei: study of its possible role as C1 factor of enzymatic lignocellulose conversion. Biotechnol. Biofuels 9:178. 10.1186/s13068-016-0590-2 27570542 PMC5000517

[B34] Fuller K. K. Dunlap J. C. Loros J. J. (2018). Light-regulated promoters for tunable, temporal, and affordable control of fungal gene expression. Appl. Mirobiol. Biotechnol. 102, 3849–3863. 10.1007/s00253-018-8887-7 29569180 PMC5897137

[B35] Furukawa T. Shida Y. Kitagami N. Mori K. Kato M. Kobayashi T.. (2009). Identification of specific binding sites for XYR1, a transcriptional activator of cellulolytic and xylanolytic genes in *Trichoderma reesei*. Fungal Genet. Biol. 46, 564–574. 10.1016/j.fgb.2009.04.001 19393758

[B36] Fussenegger M. Morris R. P. Fux C. Rimann M. Von Stockar B. Thompson C. J.. (2000). Streptogramin-based gene regulation systems for mammalian cells. Nat. Biotechnol. 18, 1203–1208. 10.1038/81208 11062442

[B37] Gagniuc P. Ionescu-Tirgoviste C. Levine M. Tjian R. Smale S. Kadonaga J.. (2012). Eukaryotic genomes may exhibit up to 10 generic classes of gene promoters. BMC Genomics 13:512. 10.1186/1471-2164-13-512 23020586 PMC3549790

[B38] Gamauf C. Schirrmacher G. Seiboth B. Kubicek C. Bischof R. (2018). Method for Selective Carbon Source-Independent Expression of Protein-Encoding Sequences in a Filamentous Fungus Cell. European Patent No EP 3 296 401. München: European Patent Office.

[B39] Geever R. F. Baum J. A. Case M. E. Giles N. H. (1987). Regulation of the *QA* gene cluster of *Neurospora crassa*. Antonie Van Leeuwenhoek 53, 343–348. 10.1007/BF00400558 2961304

[B40] Gelev V. Zabolotny J. M. Lange M. Hiromura M. Yoo S. W. Orlando J. S.. (2014). A new paradigm for transcription factor TFIIB functionality. Sci. Rep. 4:3664. 10.1038/srep03664 24441171 PMC3895905

[B41] Godbole S. Decker S. R. Nieves R. A. Adney W. S. Vinzant T. B. Baker J. O.. (1999). Cloning and expression of *Trichoderma reesei* cellobiohydrolase I in *Pichia pastoris*. Biotechnol. Prog. 15, 828–833. 10.1021/bp9901116 10514252

[B42] Gossen M. Bujard H. (1992). Tight control of gene expression in mammalian cells by tetracycline-responsive promoters. Proc. Natl. Acad. Sci. U.S.A. 89, 5547–5551. 10.1073/pnas.89.12.5547 1319065 PMC49329

[B43] Gremel G. Dorrer M. Schmoll M. (2008). Sulphur metabolism and cellulase gene expression are connected processes in the filamentous fungus *Hypocrea jecorina* (anamorph *Trichoderma reesei*). BMC Microbiol. 8:174. 10.1186/1471-2180-8-174 18842142 PMC2584116

[B44] Gritzali M. Brown R. J. (1979). Relationships between purified extracellular enzymes from induced or cellulose-grown cells, in Hydrolysis of Cellulose: Mechanisms of Enzymatic And Acid Catalysis, eds Brown R. J. Jurasek L. 237–260. 10.1021/ba-1979-0181.ch012

[B45] Gruber F. Visser J. Kubicek C. P. De Graaff L. H. (1990). The development of a heterologous transformation system for the cellulolytic fungus *Trichoderma reesei* based on a *pyrG*-negative mutant strain. Curr. Genet. 18, 71–76. 10.1007/BF00321118 2245476

[B46] Häkkinen M. Valkonen M. J. Westerholm-Parvinen A. Aro N. Arvas M. Vitikainen M.. (2014). Screening of candidate regulators for cellulase and hemicellulase production in *Trichoderma reesei* and identification of a factor essential for cellulase production. Biotechnol. Biofuels 7:14. 10.1186/1754-6834-7-14 24472375 PMC3922861

[B47] Harkki A. Uusitalo J. Bailey M. Penttilä M. Knowles J. K. C. (1989). A novel fungal expression system: secretion of active calf chymosin from the filamentous fungus *Trichoderma reesei*. Bio/Technology 7, 596–603. 10.1038/nbt0689-596

[B48] Hartner F. S. Ruth C. Langenegger D. Johnson S. N. Hyka P. Lin-Cereghino G. P.. (2008). Promoter library designed for fine-tuned gene expression in *Pichia pastoris*. Nucleic Acids Res. 36, 1–15. 10.1093/nar/gkn369 18539608 PMC2475614

[B49] He R. Ma L. Li C. Jia W. Li D. Zhang D.. (2014). *Trpac1*, a pH response transcription regulator, is involved in cellulase gene expression in *Trichoderma reesei*. Enzyme Microb. Technol. 67, 17–26. 10.1016/j.enzmictec.2014.08.013 25442944

[B50] He R. Zhang C. Guo W. Wang L. Zhang D. Chen S. (2013). Construction of a plasmid for heterologous protein expression with a constitutive promoter in *Trichoderma reesei*. Plasmid 70, 425–429. 10.1016/j.plasmid.2013.09.004 24120481

[B51] Helmschrott C. Sasse A. Samantaray S. Krappmann S. Wagener J. (2013). Upgrading fungal gene expression on demand: improved systems for doxycycline-dependent silencing in *Aspergillus fumigatus*. Appl. Environ. Microbiol. 79, 1751–1754. 10.1128/AEM.03626-12 23275515 PMC3591957

[B52] Herold S. Bischof R. Metz B. Seiboth B. Kubicek C. P. (2013). Xylanase gene transcription in *Trichoderma reesei* is triggered by different inducers representing different hemicellulosic pentose polymers. Eukaryotic Cell 12, 390–398. 10.1128/EC.00182-12 23291620 PMC3629775

[B53] Hirasawa H. Shioya K. Furukawa T. Tani S. Sumitani J. -I. Kawaguchi T.. (2018). Engineering of the *Trichoderma reesei* xylanase3 promoter for efficient enzyme expression. Appl. Microbiol. Biotechnol. 102, 2737–2752. 10.1007/s00253-018-8763-5 29417196

[B54] Huang L. Yuan Z. Liu P. Zhou T. (2015). Effects of promoter leakage on dynamics of gene expression. BMC Syst. Biol. 9:16. 10.1186/s12918-015-0157-z 25888718 PMC4384279

[B55] Hughes R. M. Bolger S. Tapadia H. Tucker C. L. (2012). Light-mediated control of DNA transcription in yeast. Methods 58, 385–391. 10.1016/j.ymeth.2012.08.004 22922268 PMC3526682

[B56] Ilmén M. Onnela M. L. Klemsdal S. Keränen S. Penttilä M. (1996a). Functional analysis of the cellobiohydrolase I promoter of the filamentous fungus *Trichoderma reesei*. Mol. Gen. Genet. 253:303–314. 10.1007/s004380050661 9003317

[B57] Ilmén M. Thrane C. Penttilä M. (1996b). The glucose repressor gene *cre1* of *Trichoderma*: isolation and expression of a full length and a truncated mutant form. Mol. Gen. Genet. 251, 451–460. 10.1007/s004380050189 8709949

[B58] Ivanova C. Bååth J. A. Seiboth B. Kubicek C. P. (2013). Systems analysis of lactose metabolism in *Trichoderma reesei* identifies a lactose permease that is essential for cellulase induction. PLoS ONE 8:e62631. 10.1371/journal.pone.0062631 23690947 PMC3648571

[B59] Jeoh T. Michener W. Himmel M. E. Decker S. R. Adney W. S. (2008). Implications of cellobiohydrolase glycosylation for use in biomass conversion. Biotechnol. Biofuels 1:10. 10.1186/1754-6834-1-10 18471276 PMC2427024

[B60] Jørgensen T. R. Nitsche B. M. Lamers G. E. Arentshorst M. van den Hondel C. A. Ram A. F. (2010). Transcriptomic insights into the physiology of *Aspergillus niger* approaching a specific growth rate of zero. Appl. Environ. Microbiol. 76, 5344–5355. 10.1128/AEM.00450-10 20562270 PMC2918955

[B61] Juven-Gershon T. Cheng S. Kadonaga J. T. (2006). Rational design of a super core promoter that enhances gene expression. Nat. Methods 3, 917–922. 10.1038/nmeth937 17124735

[B62] Juven-Gershon T. Kadonaga J. T. (2010). Regulation of gene expression via the core promoter and the basal transcriptional machinery. Dev. Biol. 339, 225–229. 10.1016/j.ydbio.2009.08.009 19682982 PMC2830304

[B63] Karhunen T. Mäntylä A. Nevalainen K. M. H. Suominen P. L. (1993). High frequency one-step gene replacement in Trichoderma reesei. I. endoglucanase I overproduction. Mol. Gen. Genet. 241, 515–522. 10.1007/BF00279893 8264526

[B64] Kawamori M. Morikawa Y. Takasawa S. (1986). Induction and production of cellulases by l-sorbose in *Trichoderma reesei*. Appl. Microbiol. Biotechnol. 24, 449–453. 10.1007/BF00250321

[B65] Keränen S. Penttilä M. (1995). Production of recombinant proteins in the filamentous fungus *Trichoderma reesei*. Curr. Opin. Biotechnol. 6, 534–537. 10.1016/0958-1669(95)80088-3 7579664

[B66] Kiesenhofer D. P. Mach R. L. Mach-Aigner A. R. (2017). Influence of *cis* element arrangement on promoter strength in *Trichoderma reesei*. Appl. Environ. Microbiol. 84:e01742–17. 10.1128/AEM.01742-17 29079620 PMC5734013

[B67] Kubicek C. P. Mikus M. Schuster A. Schmoll M. Seiboth B. (2009). Metabolic engineering strategies for the improvement of cellulase production by *Hypocrea jecorina*. Biotechnol. Biofuels 2:19. 10.1186/1754-6834-2-19 19723296 PMC2749017

[B68] Kunitake E. Kobayashi T. (2017). Conservation and diversity of the regulators of cellulolytic enzyme genes in Ascomycete fungi. Curr. Genet. 63, 951–958. 10.1007/s00294-017-0695-6 28451846

[B69] Kurzatkowski W. Törrönen A. Filipek J. Mach R. L. Herzog P. Sowka S.. (1996). Glucose-induced secretion of *Trichoderma reesei* xylanases. Appl. Environ. Microbiol. 62, 2859–2865. 8702278 10.1128/aem.62.8.2859-2865.1996 PMC168071

[B70] Lamb T. M. Vickery J. Bell-Pedersen D. (2013). Regulation of gene expression in *Neurospora crassa* with a copper responsive promoter. G3:Genes|Genomes|Genetics 3, 2273–2280. 10.1534/g3.113.008821 24142928 PMC3852388

[B71] Landowski C. P. Huuskonen A. Wahl R. Westerholm-Parvinen A. Kanerva A. Hänninen A. L.. (2015). Enabling low cost biopharmaceuticals: a systematic approach to delete proteases from a well-known protein production host *Trichoderma reesei*. PLoS ONE 10:e0134723. 10.1371/journal.pone.0134723 26309247 PMC4550459

[B72] Le Crom S. Schackwitz W. Pennacchio L. Magnuson J. K. Culley D. E. Collett J. R.. (2009). Tracking the roots of cellulase hyperproduction by the fungus *Trichoderma reesei* using massively parallel DNA sequencing. Proc. Natl. Acad. Sci.U.S.A. 106, 16151–16156. 10.1073/pnas.0905848106 19805272 PMC2752593

[B73] Lemon B. Tjian R. (2000). Orchestrated response: a symphony of transcription factors for gene control. Genes Dev. 14, 2551–2569. 10.1101/gad.831000 11040209

[B74] Li J. Wang J. Wang S. Xing M. Yu S. Liu G. (2012). Achieving efficient protein expression in *Trichoderma reesei* by using strong constitutive promoters. Microb. Cell Fact. 11:1. 10.1186/1475-2859-11-84 22709462 PMC3439336

[B75] Li Y. Zhang X. Xiong L. Mehmood M. A. Zhao X. Bai F. (2017). On-site cellulase production and efficient saccharification of corn stover employing *cbh2* overexpressing *Trichoderma reesei* with novel induction system. Bioresour. Technol. 238, 643–649. 10.1016/j.biortech.2017.04.084 28486197

[B76] Lichius A. Bidard F. Buchholz F. Le Crom S. Martin J. Schackwitz W. (2015). Genome sequencing of the *Trichoderma reesei* QM9136 mutant identifies a truncation of the transcriptional regulator XYR1 as the cause for its cellulase-negative phenotype. BMC Genomics 16:326 10.1186/s12864-015-1526-0 25909478 PMC4409711

[B77] Lim C. Y. Santoso B. Boulay T. Dong E. Ohler U. Kadonaga J. T. (2004). The MTE, a new core promoter element for transcription by RNA polymerase II. Genes Dev. 18, 1606–1617. 10.1101/gad.1193404 15231738 PMC443522

[B78] Linger J. G. Taylor L. E. Baker J. O. Vander Wall T. Hobdey S. E. Podkaminer K.. (2015). A constitutive expression system for glycosyl hydrolase family 7 cellobiohydrolases in *Hypocrea jecorina*. Biotechnol. Biofuels 8, 1–12. 10.1186/s13068-015-0230-2 25904982 PMC4405872

[B79] Liu R. Chen L. Jiang Y. Zou G. Zhou Z. (2017). A novel transcription factor specifically regulates GH11 xylanase genes in *Trichoderma reesei*. Biotechnol. Biofuels 10:194. 10.1186/s13068-017-0878-x 28785310 PMC5541735

[B80] Liu T. Wang T. Li X. Liu X. (2008). Improved heterologous gene expression in *Trichoderma reesei* by cellobiohydrolase I gene (*cbh1*) promoter optimization. Acta Biochim. Biophys. Sin. 40, 158–165. 10.1111/j.1745-7270.2008.00388.x 18235978

[B81] Lombard V. Golaconda Ramulu H. Drula E. Coutinho P. M. Henrissat B. (2014). The carbohydrate-active enzymes database (CAZy) in 2013. Nucleic Acids Res. 42, D490–D495. 10.1093/nar/gkt1178 24270786 PMC3965031

[B82] Lv X. Zheng F. Li C. Zhang W. Chen G. Liu W. (2015). Characterization of a copper responsive promoter and its mediated overexpression of the xylanase regulator 1 results in an induction-independent production of cellulases in *Trichoderma reesei*. Biotechnol. Biofuels 8:67. 10.1186/s13068-015-0249-4 25926888 PMC4413991

[B83] Ma S. Preims M. Piumi F. Kappel L. Seiboth B. Record E.. (2017). Molecular and catalytic properties of fungal extracellular cellobiose dehydrogenase produced in prokaryotic and eukaryotic expression systems. Microb. Cell Fact. 16, 1–14. 10.1186/s12934-017-0653-5 28245812 PMC5331742

[B84] Mach R. L. Strauss J. Zeilinger S. Schindler M. Kubicek C. P. (1996). Carbon catabolite repression of xylanase I (*xyn1*) gene expression in *Trichoderma reesei*. Mol. Microbiol. 21, 1273–1281. 10.1046/j.1365-2958.1996.00094.x 8898395

[B85] Mach R. L. Zeilinger S. (2003). Regulation of gene expression in industrial fungi: *Trichoderma*. Appl. Microbiol. Biotechnol. 60, 515–522. 10.1007/s00253-002-1162-x 12536250

[B86] Mach-Aigner A. R. Gudynaite-Savitch L. Mach R. L. (2011). l-Arabitol is the actual inducer of xylanase expression in *Hypocrea jecorina* (*Trichoderma reesei*). Appl. Environ. Microbiol. 77, 5988–5994. 10.1128/AEM.05427-11 21742908 PMC3165376

[B87] Mach-Aigner A. R. Pucher M. E. Mach R. L. (2010). d-Xylose as a repressor or inducer of xylanase expression in *Hypocrea jecorina* (*Trichoderma reesei*). Appl. Env. Microbiol. 76, 1770–1776. 10.1128/Aem.02746-09 20097821 PMC2838004

[B88] Mandels M. Parrish F. W. Reese E. T. (1962). Sophorose as an inducer of cellulase in *Trichoderma viride*. J. Bacteriol. 83, 400–408. 14469205 10.1128/jb.83.2.400-408.1962 PMC277742

[B89] Mandels M. Reese E. T. (1957). Induction of cellulase in *Trichoderma viride* as influenced by carbon sources and metals. J. Bacteriol. 73, 269–278. 10.1002/path.1700730133 13416182 PMC289787

[B90] Mantovani R. (1998). A survey of 178 NF-Y binding CCAAT boxes. Nucleic Acids Res. 26, 1135–1143. 10.1093/nar/26.5.1135 9469818 PMC147377

[B91] Mao X. Hu Y. Liang C. Lu C. (2002). *MET3* promoter: a tightly regulated promoter and its application in construction of conditional lethal strain. Curr. Microbiol. 45, 37–40. 10.1007/s00284-001-0046-0 12029525

[B92] Margolles-Clark E. Tenkanen M. Nakari-Setälä T. Penttilä M. (1996). Cloning of genes encoding α-l-arabinofuranosidase and β-xylosidase from *Trichoderma reesei* by expression in *Saccharomyces cerevisiae*. Appl Env. Microbiol 62, 3840–3846. 8837440 10.1128/aem.62.10.3840-3846.1996 PMC168192

[B93] Mellitzer A. Ruth C. Gustafsson C. Welch M. Birner-Grünberger R. Weis R.. (2014). Synergistic modular promoter and gene optimization to push cellulase secretion by *Pichia pastoris* beyond existing benchmarks. J. Biotechnol. 191, 187–195. 10.1016/j.jbiotec.2014.08.035 25193713

[B94] Mello-de-Sousa T. M. Rassinger A. Derntl C. Poças-Fonseca M. J. Mach R. L. Mach-Aigner A. R. (2016). The relation between promoter chromatin status, Xyr1 and cellulase expression in *Trichoderma reesei*. Curr. Genomics 17, 145–152. 10.2174/1389202917666151116211812 27226770 PMC4864836

[B95] Mello-de-Sousa T. M. Rassinger A. Pucher M. E. dos Santos Castro L. Persinoti G. F. Silva-Rocha R.. (2015). The impact of chromatin remodelling on cellulase expression in *Trichoderma reesei*. BMC Genomics 16, 1–11. 10.1186/s12864-015-1807-7 26248555 PMC4528718

[B96] Metz B. Seidl-Seiboth V. Haarmann T. Kopchinskiy A. Lorenz P. Seiboth B.. (2011). Expression of biomass-degrading enzymes is a major event during conidium development in *Trichoderma reesei*. Eukaryotic Cell 10, 1527–1535. 10.1128/EC.05014-11 21890820 PMC3209057

[B97] Meyer V. Wanka F. van Gent J. Arentshorst M. van den Hondel C. A. Ram A. F. (2011). Fungal gene expression on demand: an inducible, tunable, and metabolism-independent expression system for *Aspergillus niger*. Appl. Environ. Microbiol. 77, 2975–2983. 10.1128/AEM.02740-10 21378046 PMC3126388

[B98] Miyauchi S. Te'o V. S. Bergquist P. L. Nevalainen K. M. H. (2013). Expression of a bacterial xylanase in *Trichoderma reesei* under the *egl2* and *cbh2* glycosyl hydrolase gene promoters. N. Biotechnol. 30, 523–530. 10.1016/j.nbt.2013.02.005 23467195

[B99] Montenecourt B. S. Eveleigh D. E. (1979). Semiquantitative plate assay for determination of cellulase production by *Trichoderma viride*. Appl. Environ. Microbiol. 33, 178–183. 16345184 10.1128/aem.33.1.178-183.1977 PMC170619

[B100] Müller F. Tora L. (2014). Chromatin and DNA sequences in defining promoters for transcription initiation. Biochim. Biophys. Acta - Gene Regul. Mech. 1839, 118–128. 10.1016/j.bbagrm.2013.11.003 24275614

[B101] Mullick A. Xu Y. Warren R. Koutroumanis M. Guilbault C. Broussau S.. (2006). The cumate gene-switch: a system for regulated expression in mammalian cells. BMC Biotechnol. 6:43. 10.1186/1472-6750-6-43 17083727 PMC1654148

[B102] Nakari T. Alatalo E. Penttilä M. E. (1993). Isolation of *Trichoderma reesei* genes highly expressed on glucose-containing media: characterization of the *tef1* gene encoding translation elongation factor 1α. Gene 136, 313–318. 10.1016/0378-1119(93)90486-M 8294023

[B103] Nakari-Setälä T. Paloheimo M. Kallio J. Vehmaanperä J. Penttilä M. Saloheimo M. (2009). Genetic modification of carbon catabolite repression in *Trichoderma reesei* for improved protein production. Appl. Environ. Microbiol. 75, 4853–4860. 10.1128/AEM.00282-09 19447952 PMC2708423

[B104] Nakari-Setälä T. Penttilä M. (1995). Production of *Trichoderma reesei* cellulases glucose-containing media. Appl. Environ. Microbiol. 61, 3650–3655. 7487002 10.1128/aem.61.10.3650-3655.1995 PMC167665

[B105] Nakazawa H. Kawai T. Ida N. Shida Y. Kobayashi Y. Okada H.. (2012). Construction of a recombinant *Trichoderma reesei* strain expressing *Aspergillus aculeatus* β-glucosidase 1 for efficient biomass conversion. Biotechnol. Bioeng. 109, 92–99. 10.1002/bit.23296 21830204

[B106] Nazar R. N. Chen P. Dean D. Robb J. (2010). DNA chip analysis in diverse organisms with unsequenced genomes. Mol. Biotechnol. 44, 8–13. 10.1007/s12033-009-9212-6 19757211

[B107] Nevalainen H. Peterson R. (2014). Making recombinant proteins in filamentous fungi - Are we expecting too much? Front. Microbiol. 5:75. 10.3389/fmicb.2014.00075 24578701 PMC3936196

[B108] Nevalainen K. M. H. Te'o V. S. J. Bergquist P. L. (2005). Heterologous protein expression in filamentous fungi. Trends Biotechnol. 23, 468–474. 10.1016/j.tibtech.2005.06.002 15967521

[B109] Nogawa M. Goto M. Okada H. Morikawa Y. (2001). l-Sorbose induces cellulase gene transcription in the cellulolytic fungus *Trichoderma reesei*. Curr. Genet. 38, 329–334. 10.1007/s002940000165 11270575

[B110] Novina C. D. Roy A. L. (1996). Core promoters and transcriptional control. Trends Genet. 12, 351–355. 10.1016/0168-9525(96)10034-2 8855664

[B111] Nummi M. Niku-Paavola M -L. Lappalainen A. Enari T.-M. Raunio V. (1983). Cellobiohydrolase from *Trichoderma reesei*. Biochem. J. 215, 677–683. 10.1042/bj2150677 6419730 PMC1152451

[B112] Ory J. J. Griffith C. L. Doering T. L. (2004). An efficiently regulated promoter system for *Cryptococcus neoformans* utilizing the *CTR4* promoter. Yeast 21, 919–926. 10.1002/yea.1139 15334556

[B113] Ottoz D. S. M. Rudolf F. Stelling J. (2014). Inducible, tightly regulated and growth condition-independent transcription factor in *Saccharomyces cerevisiae*. Nucleic Acids Res. 42:130. 10.1093/nar/gku616 25034689 PMC4176152

[B114] Pachlinger R. Mitterbauer R. Adam G. Strauss J. (2005). Metabolically independent and accurately adjustable *Aspergillus* sp. expression system. Appl. Environ. Microbiol. 71, 672–678. 10.1128/AEM.71.2.672-678.2005 15691916 PMC546773

[B115] Paloheimo M. Haarmann T. Mäkinen S. Vehmaanperä J. (2016). Production of industrial enzymes in *Trichoderma reesei*, in Gene Expression Systems in Fungi: Advancements and Applications, eds Schmoll M. Dattenböck C. (Cham: Springer), 23–58. 10.1007/978-3-319-27951-0

[B116] Penttilä M. Nevalainen H. Rättö M. Salminen E. Knowles J. K. C. (1987). A versatile transformation system for the cellulolytic filamentous fungus *Trichoderma reesei*. Gene 61, 155–164. 3127274 10.1016/0378-1119(87)90110-7

[B117] Penttilä M. E. André L. Lehtovaara P. Bailey M. Teeri T. T. Knowles J. K. C. (1988). Efficient secretion of two fungal cellobiohydrolases by *Saccharomyces cerevisiae*. Gene 63, 103–112. 10.1016/0378-1119(88)90549-5 3290051

[B118] Peterson R. Nevalainen H. (2012). *Trichoderma reesei* RUT-C30–thirty years of strain improvement. Microbiology 158, 58–68. 10.1099/mic.0.054031-0 21998163

[B119] Pierrat B. Heery D. M. Lemoine Y. Losson R. (1992). Functional analysis of the human estrogen receptor using a phenotypic transactivation assay in yeast. Gene 119, 237–245. 10.1016/0378-1119(92)90277-V 1398105

[B120] Portnoy T. Margeot A. Linke R. Atanasova L. Fekete E. Sándor E.. (2011a). The CRE1 carbon catabolite repressor of the fungus *Trichoderma reesei*: a master regulator of carbon assimilation. BMC Genomics 12:269. 10.1186/1471-2164-12-269 21619626 PMC3124439

[B121] Portnoy T. Margeot A. Seidl-Seiboth V. Le Crom S. Chaabane F. Ben Linke R.. (2011b). Differential regulation of the cellulase transcription factors XYR1, ACE2, and ACE1 in *Trichoderma reesei* strains producing high and low levels of cellulase. Eukaryotic Cell 10, 262–271. 10.1128/EC.00208-10 21169417 PMC3067402

[B122] Pucher M. E. Steiger M. G. Mach R. L. Mach-Aigner A. R. (2011). A modified expression of the major hydrolase activator in *Hypocrea jecorina* (*Trichoderma reesei*) changes enzymatic catalysis of biopolymer degradation. Catal. Today 167, 122–128. 10.1016/j.cattod.2010.12.038 27667900 PMC4461149

[B123] Quintero M. J. Maya D. Arévalo-Rodríguez M. Cebolla A. Chávez S. (2007). An improved system for estradiol-dependent regulation of gene expression in yeast. Microb. Cell Fact. 6:10. 10.1186/1475-2859-6-10 17374163 PMC1831787

[B124] Rahman Z. Shida Y. Furukawa T. Suzuki Y. Okada H. Ogasawara W.. (2009). Evaluation and characterization of *Trichoderma reesei* cellulase and xylanase promoters. Appl. Microbiol. Biotechnol. 82, 899–908. 10.1007/s00253-008-1841-3 19148637

[B125] Rantasalo A. Landowski C. P. Kuivanen J. Korppoo A. Reuter L. Koivistoinen O.. (2018). A universal gene expression system for fungi. Nucleic Acids Res. gky. [Epub ahead of print]. 10.1093/nar/gky558 29924368 PMC6182139

[B126] Reese E. T. (1975). Enzyme systems for cellulose. Biotechnol. Bioeng. Symp. 5, 77–80. 1191752

[B127] Reese E. T. (1976). History of the cellulase program at the U.S. Natick Development Center. Biotechnol. Bioeng. Symp. 6, 9–20. 826291

[B128] Reeves A. R. Weber G. Cernota W. H. Weber J. M. (2002). Analysis of an 8.1-kb DNA fragment contiguous with the erythromycin gene cluster of *Saccharopolyspora erythraea* in the *eryCI*-flanking region. Antimicrob. Agents Chemother. 46, 3892–3899. 10.1128/AAC.46.12.3892-3899.2002 12435693 PMC132777

[B129] Roeder R. G. (1996). The role of general initiation factors in transcription by RNA polymerase II. Trends Biochem. Sci. 21, 327–335. 10.1016/0968-0004(96)10050-5 8870495

[B130] Roy A. L. Singer D. S. (2015). Core promoters in transcription: old problem, new insights. Trends Biochem. Sci. 40, 165–171. 10.1016/j.tibs.2015.01.007 25680757 PMC4340783

[B131] Ruijter G. J. G. Visser J. (1997). Carbon repression in Aspergilli. FEMS Microbiol. Lett. 151:103–114. 10.1016/S0378-1097(97)00161-4 9228741

[B132] Saloheimo A. Aro N. Ilmén M. Penttilä M. (2000). Isolation of the *ace1* gene encoding a Cys2-His2 transcription factor involved in regulation of activity of the cellulase promoter *cbh1* of *Trichoderma reesei*. J. Biol. Chem. 275, 5817–5825. 10.1074/jbc.275.8.5817 10681571

[B133] Saravanakumar K. Kathiresan K. (2014). Bioconversion of lignocellulosic waste to bioethanol by *Trichoderma* and yeast fermentation. 3 Biotech 4, 493–499. 10.1007/s13205-013-0179-4 28324381 PMC4162899

[B134] Schlabach M. R. Hu J. K. Li M. Elledge S. J. (2010). Synthetic design of strong promoters. Proc. Natl. Acad. Sci.U.S.A. 107, 2538–2543. 10.1073/pnas.0914803107 20133776 PMC2823900

[B135] Seiboth B. Karimi R. A. Phatale P. A. Linke R. Hartl L. Sauer D. G.. (2012). The putative protein methyltransferase LAE1 controls cellulase gene expression in *Trichoderma reesei*. Mol. Microbiol. 84, 1150–1164. 10.1111/j.1365-2958.2012.08083.x 22554051 PMC3370264

[B136] Seidl V. Gamauf C. Druzhinina I. S. Seiboth B. Hartl L. Kubicek C. P. (2008). The *Hypocrea jecorina* (*Trichoderma reesei*) hypercellulolytic mutant RUT C30 lacks a 85 kb (29 gene-encoding) region of the wild-type genome. BMC Genomics 9:327. 10.1186/1471-2164-9-327 18620557 PMC2483294

[B137] Seidl V. Seiboth B. (2010). *Trichoderma reesei*: genetic approaches to improving strain efficiency. Biofuels 1, 343–354. 10.4155/bfs.10.1

[B138] Shoji J. Y. Maruyama J. I. Arioka M. Kitamoto K. (2005). Development of *Aspergillus oryzae thiA* promoter as a tool for molecular biological studies. FEMS Microbiol. Lett. 244, 41–46. 10.1016/j.femsle.2005.01.014 15727819

[B139] Smale S. T. Kadonaga J. T. (2003). The RNA polymerase II core promoter. Annu. Rev. Biochem. 72, 449–479. 10.1146/annurev.biochem.72.121801.161520 12651739

[B140] Steiger M. G. (2013). Molecular tools in *Trichoderma* genetic studies, in Trichoderma: Biology and Applications, eds Mukherjee P. K. Horwitz B. A. Shankar Shingh U. Mukherjee M. Schmoll M. (Wallingford: CABI International), 128–143. 10.1079/9781780642475.0128

[B141] Strauss J. Mach R. L. Zeilinger S. Hartler G. Stöffler G. Wolschek M.. (1995). Cre1, the carbon catabolite repressor protein from *Trichoderma reesei*. FEBS Lett. 376, 103–107. 10.1016/0014-5793(95)01255-5 8521952

[B142] Stricker A. R. Grosstessner-Hain K. Würleitner E. Mach R. L. (2006). Xyr1 (Xylanase Regulator 1) regulates both the hydrolytic enzyme system and d-xylose metabolism in *Hypocrea jecorina*. Eukaryotic Cell 5, 2128–2137. 10.1128/EC.00211-06 17056741 PMC1694815

[B143] Stricker A. R. Mach R. L. De Graaff L. H. (2008a). Regulation of transcription of cellulases- and hemicellulases-encoding genes in *Aspergillus niger* and *Hypocrea jecorina* (*Trichoderma reesei*). Appl. Microbiol. Biotechnol. 78, 211–220. 10.1007/s00253-007-1322-0 18197406

[B144] Stricker A. R. Trefflinger P. Aro N. Penttilä M. Mach R. L. (2008b). Role of Ace2 (Activator of Cellulases 2) within the *xyn2* transcriptosome of *Hypocrea jecorina*. Fungal Genet. Biol. 45, 436–445. 10.1016/j.fgb.2007.08.005 17920314

[B145] Suto M. Tomita F. (2001). Induction and catabolite repression mechanisms of cellulase in fungi. J. Biosci. Bioeng. 92, 305–311. 10.1016/S1389-1723(01)80231-0 16233102

[B146] Svejstrup J. Q. (2004). The RNA polymerase II transcription cycle: cycling through chromatin. Biochim. Biophys. Acta - Gene Struct. Expr. 1677, 64–73. 10.1016/j.bbaexp.2003.10.012 15020047

[B147] Takashima S. Iikura H. Nakamura A. Masaki H. Uozumi T. (1996). Analysis of Cre1 binding sites in the *Trichoderma reesei cbh1* upstream region. FEMS Microbiol. Lett. 145, 361–366. 10.1016/S0378-1097(96)00432-6 8978090

[B148] Till P. Pucher M. E. Mach R. L. Aigner A. R. M. (2018). A long noncoding RNA promotes cellulase expression in *Trichoderma reesei*. Biotechnol. Biofuels 11:78. 10.1186/s13068-018-1081-4 29588663 PMC5865335

[B149] Tisch D. Kubicek C. P. Schmoll M. (2011). The phosducin-like protein PhLP1 impacts regulation of glycoside hydrolases and light response in *Trichoderma reesei*. BMC Genomics 12:61. 10.1186/1471-2164-12-613 22182583 PMC3267782

[B150] Uzbas F. Sezerman U. Hartl L. Kubicek C. P. Seiboth B. (2012). A homologous production system for *Trichoderma reesei* secreted proteins in a cellulase-free background. Appl. Microbiol. Biotechnol. 93, 1601–1608. 10.1007/s00253-011-3674-8 22080343 PMC3275749

[B151] Vaheri M. P. Vaheri M. E. O. Kauppinen V. S. (1979). Formation and release of cellulolytic enzymes during growth of *Trichoderma reesei* on cellobiose and glycerol. Eur. J. Appl. Microbiol. Biotechnol. 8, 73–80. 10.1007/BF00510268

[B152] van Arsdell J. N. Kwok S. Schweickart V. L. Ladner M. B. Gelfand D. H. Innis M. A. (1987). Cloning, characterization and expression in *Saccharomyces cerevisiae* of endoglucanase I from *Trichoderma reesei*. Nat. Biotechnol. 5, 60–64.

[B153] Vogl T. Ruth C. Pitzer J. Kickenweiz T. Glieder A. (2014). Synthetic core promoters for *Pichia pastoris*. ACS Synth. Biol. 3, 188–191. 10.1021/sb400091p 24187969 PMC3964828

[B154] Vogt K. Bhabhra R. Rhodes J. C. Askew D. S. (2005). Doxycycline-regulated gene expression in the opportunistic fungal pathogen *Aspergillus fumigatus*. BMC Microbiol. 5:1. 10.1186/1471-2180-5-1 15649330 PMC546209

[B155] Wang L. Zheng F. Zhang W. Zhong Y. Chen G. Meng X. (2018). A copper-controlled RNA interference system for reversible silencing of target genes in *Trichoderma reesei*. Biotechnol. Biofuels 11:33 10.1186/s13068-018-1038-7 29449881 PMC5806297

[B156] Wang M. Lu X. (2016). Exploring the synergy between cellobiose dehydrogenase from *Phanerochaete chrysosporium* and cellulase from *Trichoderma reesei*. Front. Microbiol. 7:620. 10.3389/fmicb.2016.00620 27199949 PMC4850161

[B157] Wang W. Shi X.-Y. Wei D.-Z. (2014). Light-mediated control of gene expression in filamentous fungus *Trichoderma reesei*. J. Microbiol. Methods 103, 37–39. 10.1016/j.mimet.2014.05.017 24886835

[B158] Wang Z. Gerstein M. Snyder M. (2009). RNA-Seq: a revolutionary tool for transcriptomics. Nat. Rev. Genet. 10, 57–63. 10.1038/nrg2484 19015660 PMC2949280

[B159] Wanka F. Arentshorst M. Cairns T. C. Jørgensen T. Ram A. F. J. Meyer V. (2016a). Highly active promoters and native secretion signals for protein production during extremely low growth rates in *Aspergillus niger*. Microb. Cell Fact. 15, 1–12. 10.1186/s12934-016-0543-2 27544686 PMC4992228

[B160] Wanka F. Cairns T. Boecker S. Berens C. Happel A. Zheng X.. (2016b). Tet-on, or Tet-off, that is the question : advanced conditional gene expression in *Aspergillus*. Fungal Genet. Biol. 89, 72–83. 10.1016/j.fgb.2015.11.003 26555930

[B161] Ward M. (2011). Trichoderma Promoter. U.S. Patent No. US8044192. Washington, DC: U.S. Patent and Trademark Office.

[B162] Ward O. P. (2012). Production of recombinant proteins by filamentous fungi. Biotechnol. Adv. 30, 1119–1139. 10.1016/j.biotechadv.2011.09.012 21968147

[B163] Weber W. Lienhart C. Daoud-El Baba M. Fussenegger M. (2009). A biotin-triggered genetic switch in mammalian cells and mice. Metab. Eng. 11, 117–124. 10.1016/j.ymben.2008.12.001 19271268

[B164] Weber W. Marty R. R. Link N. Ehrbar M. Keller B. Weber C. C.. (2003). Conditional human VEGF-mediated vascularization in chicken embryos using a novel temperature-inducible gene regulation (TIGR) system. Nucleic Acids Res. 31:e69. 10.1093/nar/gng069 12799458 PMC162344

[B165] Wei W. Pelechano V. Järvelin A. I. Steinmetz L. M. (2011). Functional consequences of bidirectional promoters. Trends Genet. 27, 267–276. 10.1016/j.tig.2011.04.002 21601935 PMC3123404

[B166] Weis L. Reinberg D. (1997). Accurate positioning of RNA polymerase II on a natural TATA-less promoter is independent of TATA-binding-protein-associated factors and initiator-binding proteins. Mol. Cell. Biol. 17, 2973–2984. 10.1128/MCB.17.6.2973 9154795 PMC232149

[B167] Xu J. Nogawa M. Okada H. Morikawa Y. (2000). Regulation of *xyn3* gene expression in *Trichoderma reesei* PC-3-7. Appl. Microbiol. Biotechnol. 54, 370–375. 10.1007/s002530000410 11030574

[B168] Xu Q. Himmel M. E. Singh A. (2015). Production of ethanol from engineered *Trichoderma reesei*, in Direct Microbial Conversion of Biomass to Advanced Biofuels, ed. Himmel M. E. (Amsterdam: Elsevier), 197–208.

[B169] Xuan Y. Zhou X. Zhang W. Zhang X. Song Z. Zhang Y. (2009). An upstream activation sequence controls the expression of *AOX1* gene in *Pichia pastoris*. FEMS Yeast Res. 9, 1271–1282. 10.1111/j.1567-1364.2009.00571.x 19788557

[B170] Yella V. R. Bansal M. (2017). DNA structural features of eukaryotic TATA-containing and TATA-less promoters. FEBS Open Bio 7, 324–334. 10.1002/2211-5463.12166 28286728 PMC5337902

[B171] Zeilinger S. Ebner A. Marosits T. Mach R. Kubicek C. P. (2001). The *Hypocrea jecorina* HAP 2/3/5 protein complex binds to the inverted CCAAT-box (ATTGG) within the *cbh2* (cellobiohydrolase II-gene) activating element. Mol. Genet. Genomics 266, 56–63. 10.1007/s004380100518 11589578

[B172] Zeilinger S. Mach R. L. Kubicek C. P. (1998). Two adjacent protein binding motifs in the *cbh2* (cellobiohydrolase II- encoding) promoter of the fungus *Hypocrea jecorina* (*Trichoderma reesei*) cooperate in the induction by cellulose. J. Biol. Chem. 273, 34463–34471. 10.1074/jbc.273.51.34463 9852114

[B173] Zeilinger S. Mach R. L. Schindler M. Herzog P. Kubicek C. P. (1996). Different inducibility of expression of the two xylanase genes *xyn1* and *xyn2* in *Trichoderma reesei*. J. Biol. Chem. 271, 25624–25629. 10.1074/jbc.271.41.25624 8810338

[B174] Zeilinger S. Schmoll M. Pail M. Mach R. L. Kubicek C. P. (2003). Nucleosome transactions on the *Hypocrea jecorina* (*Trichoderma reesei*) cellulase promoter *cbh2* associated with cellulase induction. Mol. Genet. Genomics 270, 46–55. 10.1007/s00438-003-0895-2 12905071

[B175] Zhang G. Liu P. Wei W. Wang X. Wei D. Wang W. (2016a). A light-switchable bidirectional expression system in filamentous fungus *Trichoderma reesei*. J. Biotechnol. 240, 85–93. 10.1016/j.jbiotec.2016.11.003 27816655

[B176] Zhang J. Wu C. Wang W. Wang W. Wei D. (2018). A versatile *Trichoderma reesei* expression system for the production of heterologous proteins. Biotechnol. Lett. 40, 965–972. 10.1007/s10529-018-2548-x 29644559

[B177] Zhang L. Zhao X. Zhang G. Zhang J. Wang X. Zhang S.. (2016b). Light-inducible genetic engineering and control of non-homologous end-joining in industrial eukaryotic microorganisms: LML 3.0 and OFN 1.0. Sci. Rep. 6:20761. 10.1038/srep20761 26857594 PMC4746737

[B178] Zhang W. Cao Y. Gong J. Bao X. Chen G. Liu W. (2015). Identification of residues important for substrate uptake in a glucose transporter from the filamentous fungus *Trichoderma reesei*. Sci. Rep. 5:13829. 10.1038/srep13829 26345619 PMC4642563

[B179] Zhang W. Kou Y. Xu J. Cao Y. Zhao G. Shao J.. (2013). Two major facilitator superfamily sugar transporters from *Trichoderma reesei* and their roles in induction of cellulase biosynthesis. J. Biol. Chem. 288, 32861–32872. 10.1074/jbc.M113.505826 24085297 PMC3829138

[B180] Zheng F. Cao Y. Lv X. Wang L. Li C. Zhang W.. (2017). A copper-responsive promoter replacement system to investigate gene functions in *Trichoderma reesei*: a case study in characterizing SAGA genes. Appl. Microbiol. Biotechnol. 101, 2067–2078. 10.1007/s00253-016-8036-0 27942754

[B181] Zoltowski B. D. Schwerdtfeger C. Widom J. Loros J. J. Bilwes A. M. Dunlap J. C.. (2007). Conformational switching in the fungal light sensor Vivid. Science 316, 1054–1057. 10.1126/science.1137128 17510367 PMC3682417

[B182] Zou G. Shi S. Jiang Y. van den Brink J. de Vries R. P. Chen L.. (2012). Construction of a cellulase hyper-expression system in *Trichoderma reesei* by promoter and enzyme engineering. Microb. Cell Fact. 11:21. 10.1186/1475-2859-11-21 22314137 PMC3342899

[B183] Zuo J. Niu Q. W. Chua N. H. (2000). An estrogen receptor-based transactivator XVE mediates highly inducible gene expression in transgenic plants. Plant J. 24, 265–273. 10.1046/j.1365-313X.2000.00868.x 11069700

